# Determinants of the assembly and function of antibody variable domains

**DOI:** 10.1038/s41598-017-12519-9

**Published:** 2017-09-25

**Authors:** Eva Maria Herold, Christine John, Benedikt Weber, Stephan Kremser, Jonathan Eras, Carolin Berner, Sabrina Deubler, Martin Zacharias, Johannes Buchner

**Affiliations:** 1 Center for Integrated Protein Science Munich (CIPSM) at the Department Chemie, Technische Universität München, 85747 Garching, Germany; 2 Center for Integrated Protein Science Munich (CIPSM) at the Physics Department, Technische Universität München, 85747 Garching, Germany; 3 Present Address: Sanofi-Aventis GmbH, Industriepark Höchst, 65926 Frankfurt am Main, Germany; 4 Present Address: ETH Zürich, Otto-Stern-Weg 5, 8093 Zuerich, Switzerland

## Abstract

The antibody Fv module which binds antigen consists of the variable domains V_L_ and V_H_. These exhibit a conserved ß-sheet structure and comprise highly variable loops (CDRs). Little is known about the contributions of the framework residues and CDRs to their association. We exchanged conserved interface residues as well as CDR loops and tested the effects on two Fvs interacting with moderate affinities (K_D_s of ~2.5 µM and ~6 µM). While for the rather instable domains, almost all mutations had a negative effect, the more stable domains tolerated a number of mutations of conserved interface residues. Of particular importance for Fv association are V_L_P44 and V_H_L45. In general, the exchange of conserved residues in the V_L_/V_H_ interface did not have uniform effects on domain stability. Furthermore, the effects on association and antigen binding do not strictly correlate. In addition to the interface, the CDRs modulate the variable domain framework to a significant extent as shown by swap experiments. Our study reveals a complex interplay of domain stability, association and antigen binding including an unexpected strong mutual influence of the domain framework and the CDRs on stability/association on the one side and antigen binding on the other side.

## Introduction

In the humoral immune response, antigen recognition is mediated by immunoglobulins, specifically by the N-terminal variable domains of the light chain (V_L_) and of the heavy chain (V_H_) which associate non-covalently to form the so-called Fv-fragment. Three hyper-variable regions (complementarity determining regions or CDRs) in V_L_ and V_H_ comprise the residues interacting with antigens. They account for approximately 25% of the variable domains^1^. CDR-H3 (i.e., the third CDR of V_H_) is the most diverse of these six regions concerning length and amino acid sequence^2^. Apart from the CDRs, both variable domains exhibit a conserved β-barrel framework stabilized by an internal disulfide bridge^1–4^. They are composed of two ß-sheets, one with four strands (A, B, D, E) and one with six strands (A′, G, F, C, C′, C″), with the strands GFC’C involved in the V_H_/V_L_ interface. Several studies on the V_H_/V_L_ packing geometry have shown that residues within the framework as well as interface contributing residues of the CDRs can influence the interface^1–3,5,6^. 75% of the interface residues are constituted by framework ß-sheets and 25% by the CDRs (inter-strand links between GF, BC and C′C″, respectively)^1,3^. The interface contribution of the hypervariable loops comprises CDR1 and especially CDR3 residues^1,3^. In this context, the CDR-H3 can affect V_H_/V_L_ orientation due to small position re-adjustments by the varying number of interface contributing residues^3^. An additional influence by hypervariable loop residues is exhibited by the so called proximate zone, which is situated at the base of the antigen binding site, comprising residues that do not actively participate in the interface^3^. Several studies on the V_H_-V_L_ packing geometry showed that residues within the framework as well as interface contributing residues of the CDRs can influence the interface^1,3,5–7^. As the association of V_L_ and V_H_ is crucial for antigen binding^3^, understanding the underlying principles is of great importance.

For the V_H_ domain which is composed of about 125 residues and the V_L_ domain with about 110 residues, Chothia and co-workers suggested that the interface residues at positions 98, 44 and 36 in V_L_ and 103, 47, 45 and 37 in V_H_ according to Kabat numbering^8^ are conserved^1,7^. Wang and co-workers^9^ aimed at identifying amino acid networks important for V_H_ and V_L_ function by covariation analysis. This multiple sequence alignment approach investigates covariations between residues at all possible positions. This allows to reveal conserved amino acids by the correlation of the presence of one particular amino acid with the presence of a second one at a particular sequence position. In their study they included more than 2000 V-class sequences of human, mouse, cow, camel, Ilama, macaque and chicken with a bias towards human sequences (574 out of 2432). Generally, the majority of the most strongly conserved amino acids identified in this study were positioned at the V_H_/V_L_ interface^9^. For V_L_, amino acids Y36, Q37, P44, A43, L46 and F98 were found to be highly conserved, with all residues except Q37 directly in contact with V_H_. On the V_H_ side, amino acids V37, R38, G44, L45, E46, W47 and W103 could be identified in the V_H_/V_L_ interface with all residues except E46 and R38 in direct contact with V_L_. V_H_ W47 seems to be the central node based on the number and strength of its covariations with other interface residues, the same holds true for Y36 and P44 for the V_L_ domain. Further computational analyses revealed two main modes of interaction for V_H_ and V_L_ which is either characterized by a proline or a medium/large hydrophobic residue at position 44 in V_L_^7^. Concerning V_H_, W47 seems to be essential as it was previously mutated for improved solubility and stability but none of the mutations (W47L, W47R^10^) were favorable. Interface residues can also affect antigen binding^3,6,11–13^ by influencing the positioning of hypervariable loops. Additional experimental studies addressed the influence of the exchange of particular conserved amino acids on the association of V_H_ and V_L_ via the stabilities of covalently linked scFv and Fab fragments^14–18^. While these studies lay the groundwork, we are still far from a detailed and comprehensive understanding of the organization of the Fv interface. In this context, it is important to determine the affinity of the association of V_L_ and V_H_ directly. Strikingly, for the other domain interactions in IgG the K_D_ values differ by several orders of magnitude. For the C_H_3 dimer a K_D_ < 10^−10^ M was determined using SEC^19,20^. and the K_D_ for the interaction between C_H_1 and C_L_ was 6.2 µM obtained by the C_L_ induced change in intrinsic fluorescence of C_H_1^21^. This analysis is largely lacking for the V_L_/V_H_ interaction.

Here, we chose to use the V_L_ and V_H_ domains of the human monoclonal antibody 1HEZ (κ/IgM subclass) and the murine monoclonal antibody MAK33 (κ/IgG1 subclass) as well-studied model systems^20–23^ to analyze the contribution of different factors on V_L_ and V_H_ structure and function. The application of variable domains from different species and subclasses should reveal to which extent the mutation-associated effects are conserved. Their sequences contain all the conserved residues identified by Wang and coworkers except for an alanine at position 43 in MAK33 V_L,_ which is exchanged to serine and a glycine at position 44 in MAK V_H_, which is an arginine in MAK33. Since the relative importance of the conserved residues for structure, stability, association and antigen binding is not clear, we mutated every conserved residue against alanine and analyzed the properties of the variants in a comprehensive manner. Additionally, we generated two MAK33 V_L_ double point mutations (Y36A/P44A, Y36A/S43A) to investigate the potential synergistic nature of the mutation. Importantly, we focused in the analysis on the isolated variable domains and the direct influence of point mutations on their interaction, and not on Fab or scFv fragments as in previous studies^12,16,18^ to draw conclusions concerning their stabilities and antigen binding properties. Furthermore, we performed CDR exchange experiments to address the contribution of these structural elements on domain architecture.

Our results on the effects of mutations on domain structure, stability, association and antigen binding together with CDR exchange experiments reveal complex relationships between structural and functional properties within the V_L_ and V_H_ domains.

## Results

### The role of conserved residues for variable domain structure and stability

**Figure 1:**
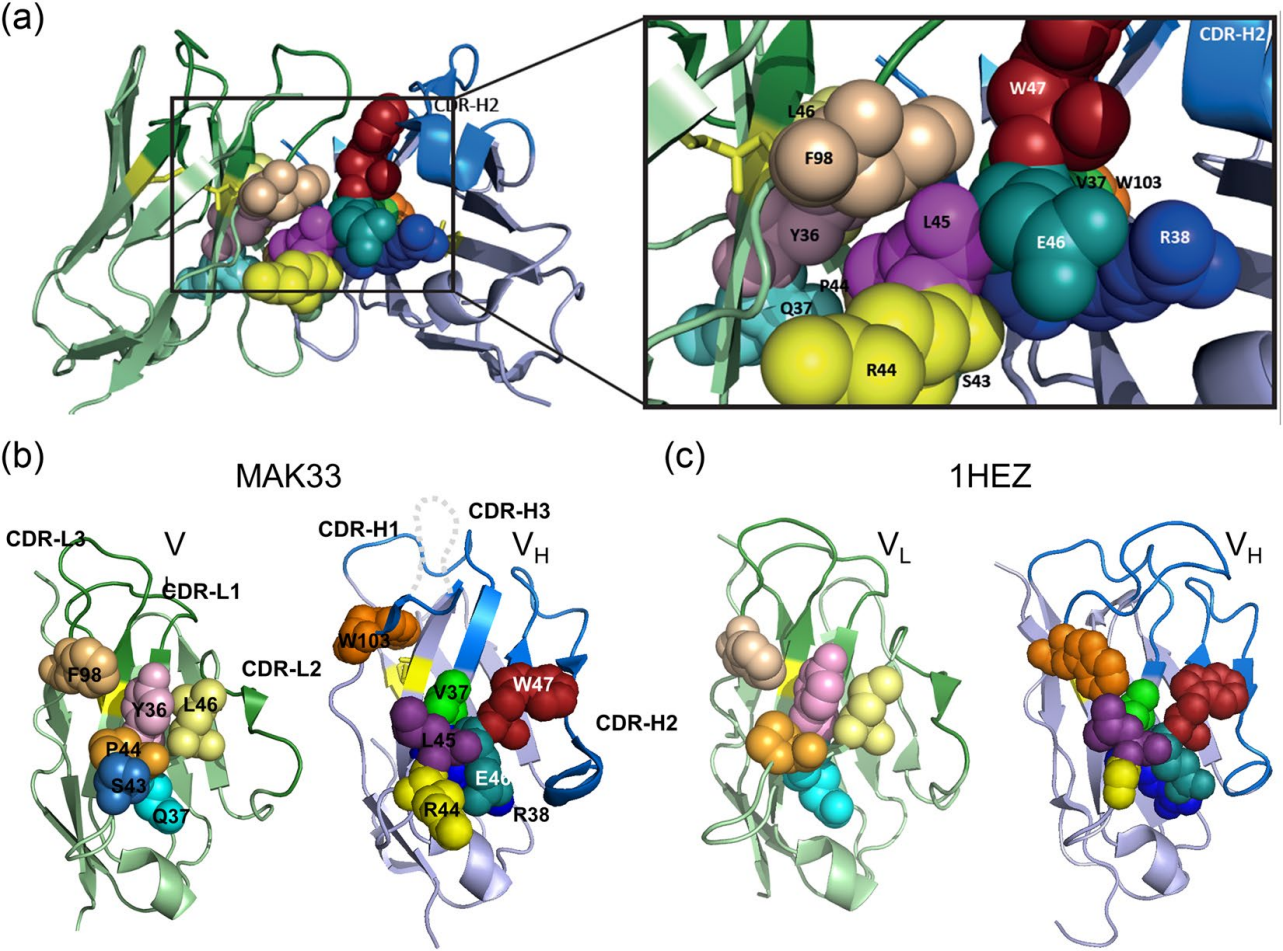
Conserved residues within the interface between V_L_ and V_H_. In (**a**) the positions of conserved amino acids in the Fv fragment of MAK33 are shown. The V_L_ domain is depicted in light green with the three CDRs highlighted in dark green. V_H_ is shown in light blue and the CDRs are in dark blue. Conserved residues within the interface are illustrated as spheres and color-coded. On the right, the region within the rectangle is enlarged. For a better orientation CDR-H2 is indicated. The labelled residues were selected for an alanine-exchange mutational analysis. In (**b**) and (**c**) the top views of the interacting residues of the V_L_ (left) and V_H_ (right) domain of MAK33 (**b**) and 1HEZ (**c**) are shown. Structures are modified from PDB ID 1FH5 and 1HEZ. The grey dotted line indicates CDR-H3 which is not resolved in PDB ID 1FH5.

**Figure 2:**
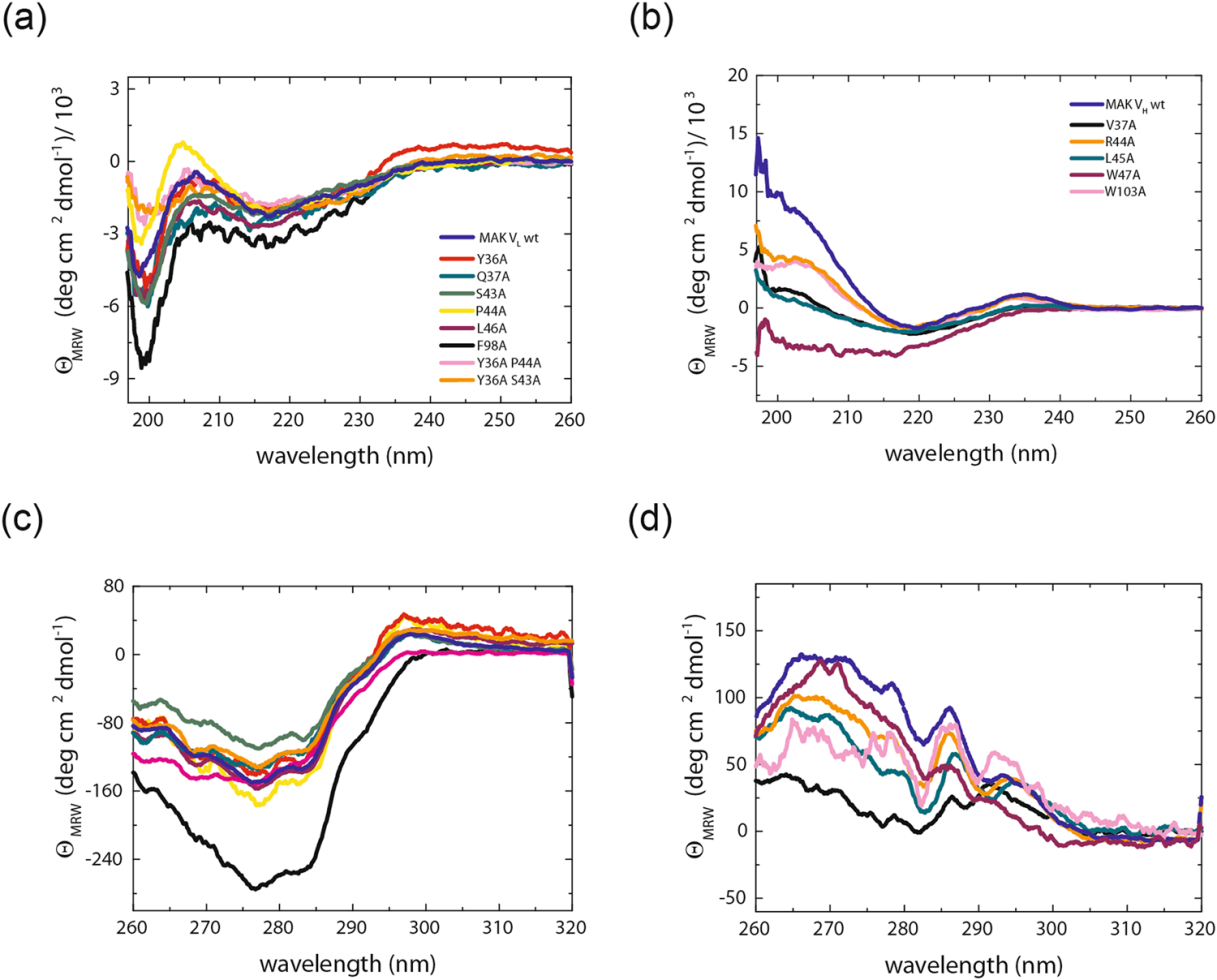
Secondary and tertiary structure of MAK33 V_L_ and V_H_ alanine exchange mutants. In (**a**) and (**b**), FUV-CD spectra of V_L_ mutants (**a**) and V_H_ mutants (**b**) are shown. Color code for V_L_: Y36A is red, Q37A dark cyan, S43A green, P44A yellow, L45A purple, F98A black, Y36A/S43A orange, Y36A/P44A pink and V_L_ wild type is royal blue. Color code for V_H_: V37A is black, R44A orange, L45A dark cyan, W47A purple, W103A pink and V_H_ wild type marine blue. In (**c**) and (**d**) the NUV-CD spectra of the V_L_ (**c**) and V_H_ (**d**) point mutants are shown. Color code for (**c**) and (**d**) is analogue to (**a**) and (**b**), respectively. For the spectra 16 accumulations each were recorded and buffer-corrected (PBS). All measurements were performed at a protein concentration of 20 µM (FUV-CD) and 50 µM (NUV-CD) in 0.5-mm (FUV) or 5-mm (NUV) quartz cuvette at 20 °C.

**Figure 3:**
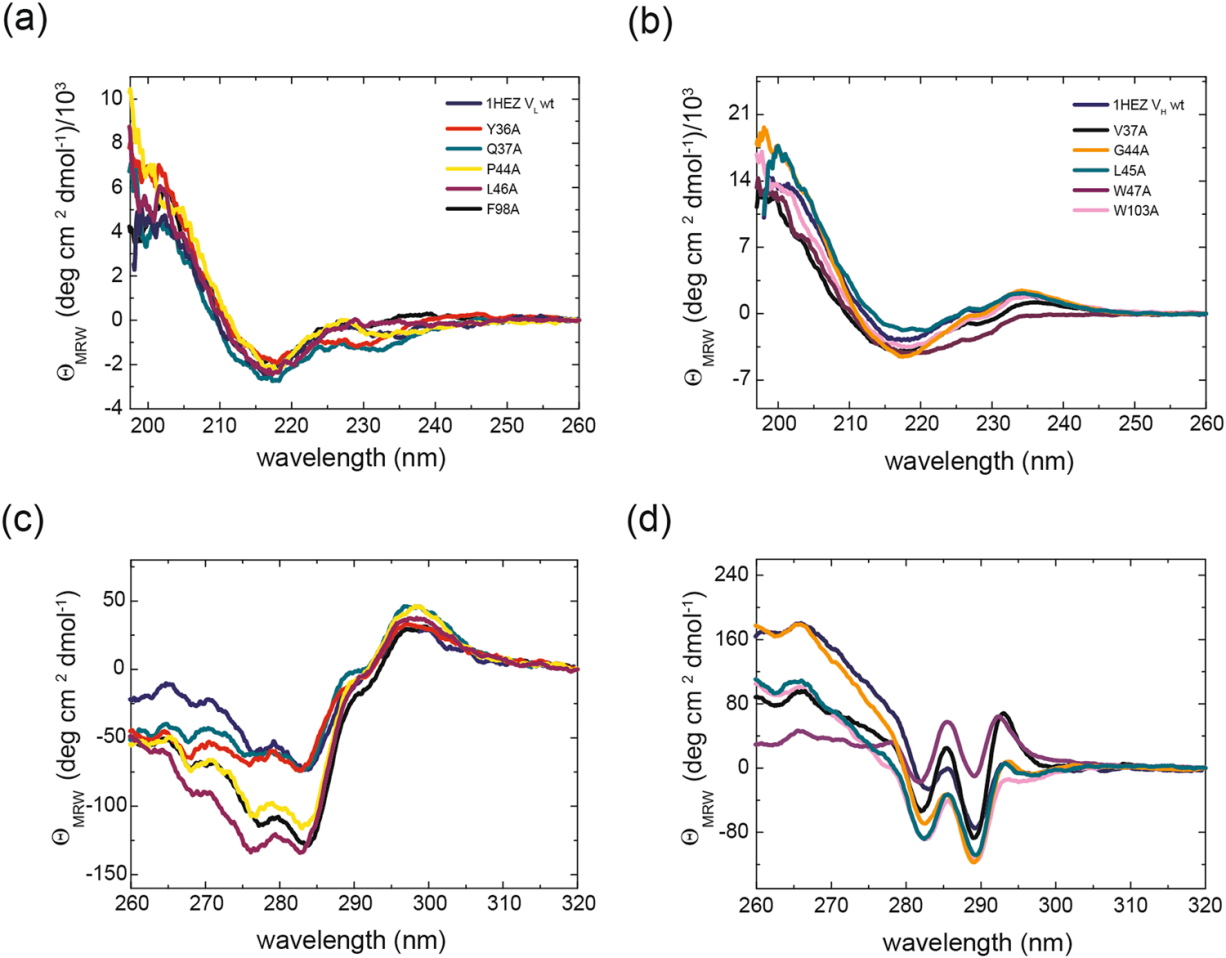
Secondary and tertiary structure of 1HEZ V_L_ and V_H_ alanine exchange mutants. In (**a**) and (**b**), FUV-CD spectra of V_L_ mutants (**a**) and V_H_ mutants (**b**) are shown. Color code for V_L_: Y36A is red, Q37A dark cyan, P44A yellow, L45A purple, F98A black, and V_L_ wild type is royal blue. Color code for V_H_: V37A is black, G44A orange, L45A dark cyan, W47A purple, W103A pink and V_H_ wild type marine blue. In (**c**) and (**d**) the NUV-CD spectra of the V_L_ (**c**) and V_H_ (**d**) point mutants are shown. Color code for (**c**) and (**d**) is analogue to (**a**) and (**b**), respectively. For the spectra 16 accumulations each were recorded and buffer-corrected (PBS). All measurements were performed at a protein concentration of 20 µM (FUV-CD) and 50 µM (NUV-CD) in 0.5-mm (FUV) or 5-mm (NUV) quartz cuvette at 20 °C.

To determine the influence of specific residues on the structure, stability, association and functionality of the individual domains, amino acids were selected for mutation which had been predicted to be important^9^. Based on the results of Wang and coworkers we created six single (Y36A, Q37A, S43A, P44A, L46A, and F98A) and two double point mutants for MAK33 V_L_ (Y36A/P44A and Y36A/S43A) and five single mutants (Y36A, Q37A, P44A, L46A, and F98A) for 1HEZ V_L_ as well as seven single point mutations for MAK33 V_H_ (V37A, R38A, R44A, L45A, E46A, W47A, and W103A) and 1HEZ V_H_ (V37A, R38A, G44A, L45A, E46A, W47A, and W103A). As shown in Fig. 1, these residues lie in or near the interaction interfaces of V_L_ and V_H_. The single domains were expressed in *E. coli*, as insoluble proteins in inclusion bodies. During refolding and purification, it turned out that R38A and E46A in V_H_ were unstable and aggregation-prone. Therefore, they were not considered further. For the determination of structural changes, we recorded CD spectra of the variants. FUV-CD spectra report on the secondary structure of proteins. All wt domains studied show a typical ß-sheet structure (minimum at 218 nm) with the exception of MAK33 V_L_, which exhibits a characteristic shape in agreement with previous studies in which we investigated the folding pathway of the protein^24^ and its amyloidogenic variants^25–27^ (Fig. 2a and b, Fig. 3a and b). In comparison to the spectra of the point mutations, a high overlap with the wt spectra is visible. Only in the case of MAK33 V_H_, the point mutants exhibited more pronounced effects on the FUV-CD spectra (Fig. 2b) including shifts in the minimum (e.g. W47A). NUV-CD spectra can be regarded as specific fingerprints for each domain. They report on the tertiary structure. Deviations in the amplitude, especially in the spectra region monitoring tyrosine and phenylalanine, were observed in the NUV spectra for some V_L_ variants: F98A for MAK33 and P44A, L46A and F98A for 1HEZ as well as for most V_H_ variants (Fig. 2c and d, Fig. 3c and d). For the V_L_ F98A variant, the different amplitude can be explained by the lack of the Phe which contributes particularly at wavelengths from 255 to 270 nm to NUV-CD spectra. Other changes in the NUV spectra are not due to the lack of aromatic amino acids since the most significant changes in the spectra are observed from 270 to 285 nm which typically indicate changes in the environment of Tyr and Trp residues. Thus, an altered tertiary structure is expected to be the origin of amplitude deviations, indicating a key structural role for these residues. In general, the CD data show that the secondary and tertiary structure of the V_L_ domain seems to tolerate single point mutations of conserved residues more than the V_H_ domain. This holds true especially for the MAK33 variable domains which exhibit major changes in the tertiary structure of the V_H_ mutants. Our results support the notion that the structure of V_H_ is generally less tolerant than V_L_ against the exchange of conserved residues.

**Figure 4:**
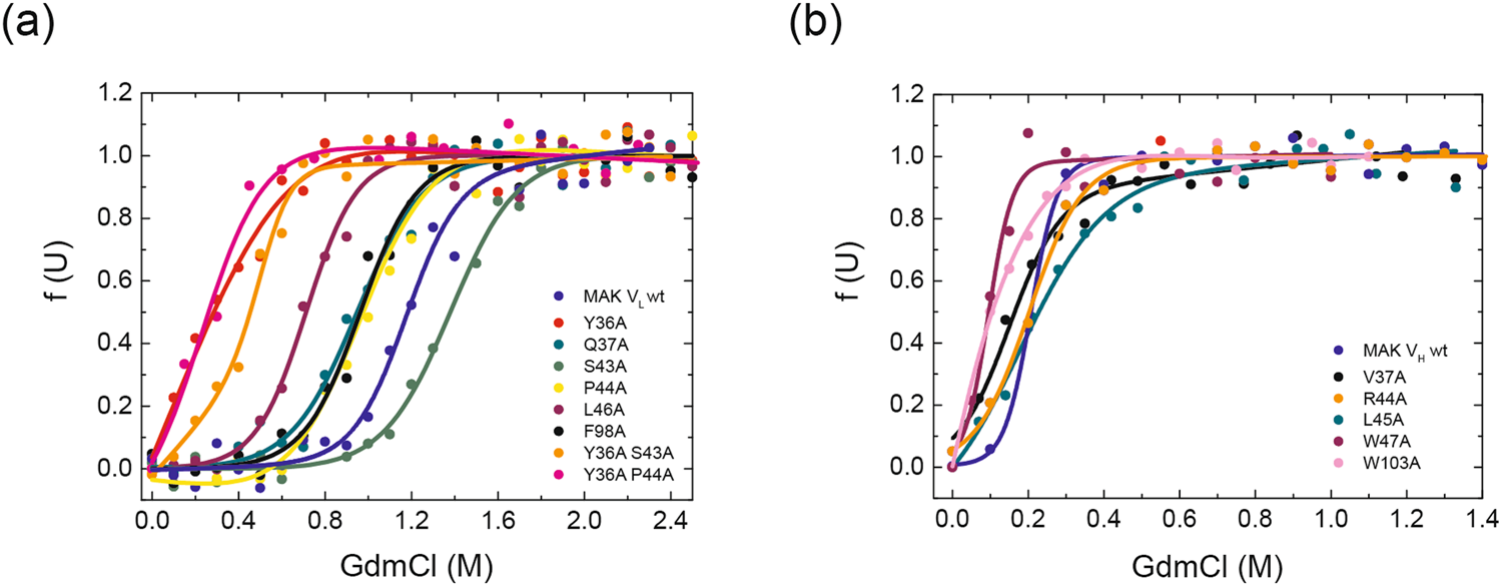
Influence of conserved residues on MAK33 protein stability. The stability of the V_H_ and V_L_ alanine point mutants towards GdmCl-induced denaturation (**a**) and (**b**). (**a**) V_L_ mutants: Y36A is red, Q37A dark cyan, S43A green, P44A yellow, L46A purple, F98A black, Y36A/S43A orange, Y36A/P44A pink and the wild type marine blue. (**b**) V_H_ mutants: V37A is black, R44A orange, L45A dark cyan, W47A purple, W103A pink and the wild type royal blue.

**Figure 5:**
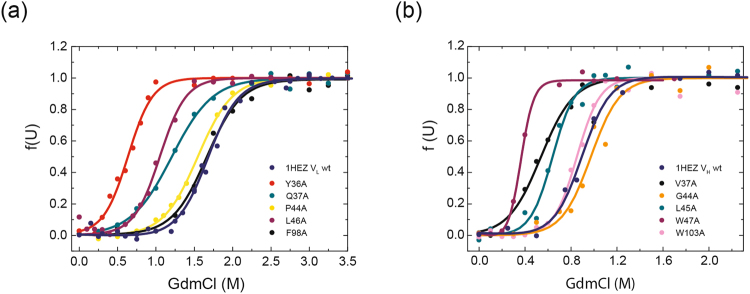
Influence of conserved residues on 1HEZ protein stability. The stability of the V_H_ and V_L_ alanine point mutants towards GdmCl-induced denaturation (**a**) and (**b**). (**a**) V_L_ mutants: Y36A is red, Q37A dark cyan, P44A yellow, L46A purple, F98A black and the wild type marine blue. (**b**) V_H_ mutants: V37A is black, G44A orange, L45A dark cyan, W47A purple, W103A pink and the wild type royal blue.

To determine whether the stability of the respective domain was affected by the mutation of conserved residues, denaturant-induced (GdmCl) unfolding transitions were performed (Figs 4 and 5, Tables 1 and 2). All transitions were fitted to a two-state model. The V_L_ domains showed a midpoint for GdmCl-induced unfolding of 1.2 ± 0.1 M GdmCl for MAK33 and 1.7 ± 0.1 M GdmCl for 1HEZ. Strikingly, MAK33 V_L_ S43A was slightly more stable than the wt against GdmCl-induced unfolding. However, the variants V_L_ Y36A and V_L_ L46A exhibited the most prominent decrease in stability compared to the two wt V_L_ domains (Figs 4 a and 5a, Tables 1 and 2). The cooperativities of these unfolding transitions are in a comparable range for all V_L_ variants, from 9.1 ± 0.8 to 23.8 ± 16.2 kJ mol^−1^ M^−1^ (Tables 1 and 2). For the least stable V_L_ point mutation Y36A, two double mutations (Y36A/P44A, Y36A/S43A) were generated in the MAK33 framework to investigate the potential synergistic nature of the observed effects. Indeed when incorporating the most stable MAK33 mutation, S43A, the stability of Y36A/S43A seemed slightly increased compared to Y36A. For Y36A/P44A, where P44A alone has wt-like stability, almost no difference in stability was visible compared to Y36A (Fig. 4a, Table 1).

**Table 1:** Characteristics of IgG (MAK33) V_H_ and V_L_ mutants.

Domain	Mutation	Association K_D_ [µM] SPR	Stability GdmCl D_1/2_ [M]	Cooperativity [kJ mol^−1^ M^−1^]	Antigen binding app. K_D_ [µM]
V_L_	**wild type**	2.0 ± 0.8*	**1.2 ± 0.1**	**17.1 ± 6.7**	**0.9 ± 0.1**
	Y36A	1.5 ± 0.2^*^	0.30 ± 0.1	16.1 ± 2.3	0.6 ± 0.1
	Q37A	1.3 ± 0.7^*^	0.9 ± 0.5	16.0 ± 5.2	1.5 ± 0.5
	S43A	1.6 ± 0.4^*^	1.4 ± 1.0	15.8 ± 8.6	1.4 ± 0.1
	P44A	58.0 ± 10.4^*^	1.0 ± 0.2	15.0 ± 3.2	10.5 ± 0.4
	L46A	0.4 ± 0.1^*^	0.7 ± 0.2	21.2 ± 3.5	4.6 ± 1.2
	F98A	5.8 ± 0.1^*^	1.0 ± 0.2	19.0 ± 2.8	8.3 ± 0.5
	Y36A/P44A	22.6 ± 4.4^*^	0.2 ± 0.1	23.8 ± 16.2	4.5 ± 0.7
	Y36A/S43A	1.5 ± 0.5^*^	0.4 ± 0.2	17.1 ± 6.0	0.7 ± 0.1
	MAK_1DH5	3.0 ± 0.3^*^	1.4 ± 0.2	15.6 ± 4.8	1.2 ± 0.3
	1DH5_MAK	42.3 ± 9.8^*^	1.3 ± 0.2	16.5 ± 1.8	NB
	1DH5_DH5	76.2 ± 12.8^*^	2.4 ± 0.2	17.1 ± 6.1	NB
V_H_	**wild type**	**2.9 ± 1.1**	**0.2 ± 0.1**	**68.6 ± 23.3**	**1.4 ± 0.1**
	V37A	6.8 ± 0.7	0.2 ± 0.1	35.3 ± 6.7	3.0 ± 0.4
	R44A	4.0 ± 2.0	0.2 ± 0.1	34.3 ± 3.7	1.5 ± 0.4
	L45A	51.0 ± 5.8	0.2 ± 0.1	21.5 ± 8.5	16.1 ± 0.9
	W47A	4.5 ± 1.3	0.1 ± 0.1	75.2 ± 19.4	NB
	W103A	6.9 ± 1.0	0.2 ± 0.1	15.3 ± 10.5	3.7 ± 0.1
	MAK_1DHU	2.5 ± 0.3	1.0 ± 0.1	4.5 ± 0.4	> 50
	1DHU_MAK	1.3 ± 0.1	1.3 ± 0.2	10.7 ± 0.9	NB
	1DHU_1DHU	± 0.5	2.5 ± 0.2	6.3 ± 3.6	NB
	MAK_MAK CDR3 1DHU	1.8 ± 0.2	0.4 ± 0.1	7.9 ± 1.4	14.7 ± 0.5

*K*_*D*_ *values were determined by SPR experiments*. Titrations of the mutant domains in a concentration range between 0–200 µM were measured with the corresponding wild type domain, which was immobilized on a CM5 chip. Experiments were performed at 20 °C; asterisks indicate that the temperature setting for the measurement was reduced to 15 °C due to low thermal stability. *Stabilities against the chemical denaturation (GdmCl) of the V*_*L*_ *and V*_*H*_ *mutants*. Even though most of the GdmCl-induced unfolding transitions were not reversible, data were evaluated according to a two-state equilibrium unfolding model to derive the midpoint of transitions (D_1/2_), as well as the cooperativity parameter (m), for a qualitative comparison of the data. *Functionality of the isolated variable domains determined by ELISA with the antigen creatine kinase*. Detection of the V_H_ and V_L_ wild type domains was possible via an introduced FLAG-tag at the C-termini. As a reference, the signals of the wild type domains vs. FLAG-tagged domains after 25 minutes of incubation were chosen. Samples were corrected for the signal of the single FLAG-tagged variable domains. Apparent K_D_ values were obtained by a Boltzmann fit. Experiments were performed at 20 °C. NB indicates that no binding could be observed.Values for wild type proteins are highlighted in bold.

**Table 2:** Characteristics of IgM (1HEZ) V_H_ and V_L_ mutants.

Domain	Mutation	Association K_D_ [µM] SPR	Stability GdmCl D_1/2_ [M]	Cooperativity [kJ mol^−1^ M^−1^]
V_L_	**wild type**	**8.2 ± 1.4**	**1.7 ± 0.1**	**12.1 ± 0.2**
	Y36A	3.8 ± 0.9	0.6 ± 0.1	15.2 ± 1.4
	Q37A	10.0 ± 0.8	1.2 ± 0.4	9.1 ± 0.8
	P44A	189.6 ± 49.2	1.5 ± 0.1	10.7 ± 0.9
	L46A	10.6 ± 0.5	1.1 ± 0.1	14.3 ± 1.4
	F98A	20.3 ± 2.8	1.7 ± 0.1	10.8 ± 1.8
V_H_	**wild type**	**4.2 ± 1.1**	**0.9 ± 0.1**	**18.9 ± 3.9**
	V37A	13.9 ± 0.4	0.5 ± 0.1	16.8 ± 9.1
	G44A	4.0 ± 0.1	1.0 ± 0.1	19.4 ± 8.7
	L45A	19.0 ± 2.0	0.6 ± 0.1	25.4 ± 8.4
	W47A	3.7 ± 1.0	0.4 ± 0.1	15.1 ± 4.7
	W103A	4.2 ± 1.3	0.9 ± 0.1	24.1 ± 10.4

*K*_*D*_ *values were determined by SPR experiments*. Titrations of the mutant domains in a concentration range between 0–200 µM were measured with the corresponding wild type domain, which was immobilized on a CM5 chip. Experiments were performed at 20 °C; *Stabilities against the chemical denaturation (GdmCl) of the V*_*L*_ *and V*_*H*_ *mutants*. Even though most of the GdmCl-induced unfolding transitions were not reversible, data were evaluated according to a two-state equilibrium unfolding model to derive the midpoint of transitions (D_1/2_), as well as the cooperativity parameter (m), for a qualitative comparison of the data.Values for wild type proteins are highlighted in bold.

For the V_H_ wt domains, we determined a midpoint for GdmCl-induced unfolding of 0.2 ± 0.1 M GdmCl for MAK33 and 0.9 ± 0.1 M GdmCl for 1HEZ. Thus, the investigated V_H_ domains are less stable than their corresponding V_L_ domains (Figs 4 and 5, Tables 1 and 2). Moreover, the isolated 1HEZ variable domains are in general more stable than those of MAK33. Interestingly, the 1HEZ V_H_ domain, wt and mutants, are dimeric in the AUC size distribution analysis (Fig. S8). The only exception was the 1HEZ point mutant W47A which exhibited a 50% equilibrium between monomer and dimer fraction. The analysis of the V_H_ MAK33 mutants showed that all mutants and wt already start to unfold in the presence of very low GdmCl-concentrations (Fig. 4b). Thus, no distinction in stability of the MAK33 V_H_ mutants can be made, assuming an equally low GdmCl tolerance for all variants. For 1HEZ V_H_, the V_H_ W47A and V_H_ V37A mutants showed the largest decrease in stability, followed by L45A (Table 2). The cooperativity of unfolding for the different MAK33 V_H_ mutations was subject to variation, from 15.3 ± 10.5 to 75.2 ± 19.4 kJ mol^−1^ M^−1^ suggesting that structural changes occurred^28^. The cooperativity values for 1HEZ V_H_, though, were in a similar range between 15.1 ± 4.7 to 24.1 ± 10.4 kJ mol^−1^M^−1^. As there is no correlation between the cooperativity values and the structural data obtained by CD measurements (Fig. 3) this assumption cannot be confirmed. In summary, MAK33 V_H_ domains exhibit relatively low stabilities compared to the other variable domains analyzed. Interestingly, MAK33 V_L_ S43A was even slightly more stable than the wt protein. For the double mutants, an additive effect on the stability could be observed. Our analysis identified W47A and V37A for V_H_ and Y36A for V_L_ as the least stable mutations. Concerning 1 HEZ V_H_, it has to be kept in mind that the wt and all mutants except for W47A (monomer dimer equilibrium) form dimers, which usually enhances its intrinsic stability. In fact, dimerization adds an additional layer of complexity. However, homo-dimerization is a typical feature of many variable antibody domains and also a number of light chains. Therefore, this additional dimension of the V_L_-V_H_ interplay was included to this study as it competes with hetero-dimerization and thus it is interesting to determine to which extent this equilibrium influences the effects of certain mutations on the interaction of the two variable dimers in the heterodimer.

### The association of V_H_ and V_L_ is particularly sensitive to mutations in V_H_

Besides influencing the structure and stability of the variable domains, the conserved residues might also play a role in the association of the two domains. In previous studies, the association of the V_L_ and V_H_ domains was analyzed using scFvs or Fab fragments^12,16,18^. This is a relatively indirect approach, as the domains were either artificially linked (scFvs) or two additional covalently linked domains were present (C_L_ and C_H_1 in the Fab). Here, we used assays that report directly on the formation of the V_L_ and V_H_ heterodimer. For SPR measurements, the wild type domain was immobilized on the chip and different concentrations of the corresponding mutant domains were added under a constant flow. With this setup, K_D_s of 2.0 ± 0.8 to 2.9 ± 1.1 µM and 4.2 ± 1.1 to 8.2 ± 1.4 µM were determined for the MAK33 and 1HEZ wild type V_L_ and V_H_ domains, respectively (Tables 1 and 2). Thus, the variable domains interact with a similar affinity as the C_H_1 and C_L_^21^ domains, but have a lower affinity compared to the C_H_3 homodimer^19,20^.

**Figure 6:**
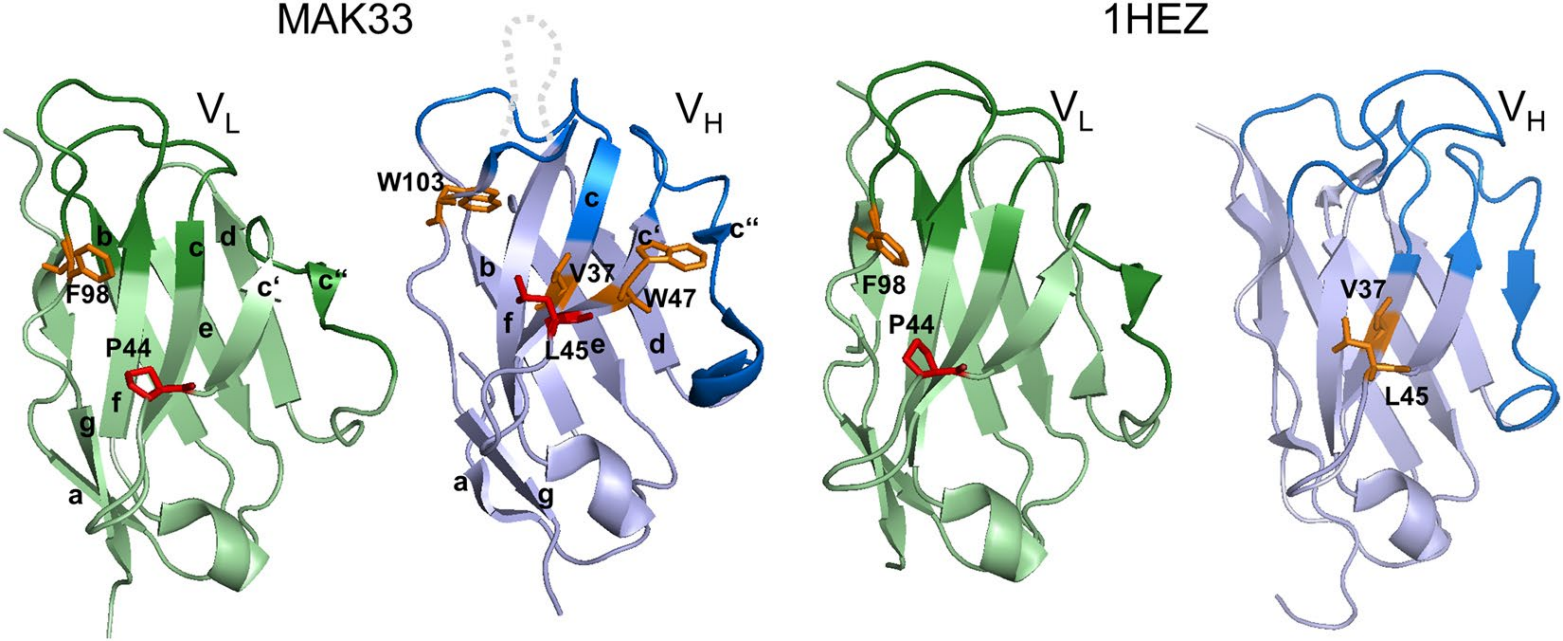
Conserved interface residues with importance for V_H_/V_L_ association. The top-views for MAK33 (left) and 1HEZ (right) V_L_ and V_H_ are shown and the residues with strong influence on association highlighted in red, those with mild influence highlighted in orange. For MAK33 the numbering of the strands (a, b, c, c′, c″, d, e, f, g) is also shown. Structures are modified from PDB ID 1FH5 and 1HEZ. The grey dotted line indicates CDR-H3 which is not resolved in PDB ID 1FH5.

Almost all MAK33 V_H_ mutants led to a decrease in the binding affinity, especially L45A with a 20-fold higher K_D_ than the wt, followed by V37A and W103A with a 2-fold change. On the 1HEZ side, only the mutants L45A and V37A exhibited a negative influence on the binding of the corresponding V_L_ wt domain. Generally, the MAK33 V_H_ mutations exerted a bigger influence on the V_H_/V_L_ interaction with a maximum 20-fold increased K_D_ compared to 1HEZ with 3 to4-fold higher K_D_s than the wt V_H_ domain (Table 1; Fig. 1c). The mutation of conserved residues in V_L_, though, showed similar effects for the two V_L_ domains from different origin. In both cases a striking binding impairment with a 30-fold higher K_D_ was observed for the mutant P44A and a slightly decreased V_H_/V_L_ interaction with a 3 fold increase in K_D_ for the mutation F98A (Fig. 6; Tables 1 and 2). Concerning the two MAK33 V_L_ double mutants (Y36A/P44A, Y36A/S43A), which were generated to investigate whether the effects observed for single mutants are additive. Y36A/S43A, which is the combination of two point mutations with no change in K_D_, leads to the expected wt-like K_D_. But the point mutation P44A with the highest K_D_ for the association with V_H_ (58.0 ± 10.4 µM), exhibited improved binding when combined with Y36A, suggesting compensatory effects (Table 1).

In conclusion, P44 exhibits a strong negative impact on the association of the V_H_ and V_L_ domain, while F98 exerts only a mild influence. However, for the two different V_H_ domains, there is no complete overlap concerning the observed effects. While three of five conserved MAK33 V_H_ residues (V37, L45, W103) showed a clear influence on the association, only the mutants V37A and L45A were affected in 1HEZ V_H_ (Fig. 6; Tables 1 and 2). Nevertheless, a negative effect of mutant 1HEZ W47A which exists as monomer and dimer cannot be excluded. Despite the wt-like K_D_, an impaired association might be compensated because in contrast to the 1HEZ wt and the other 1HEZ mutants, which are dimeric, this variant exhibited a monomer-dimer equilibrium and heterodimer formation is probably favored over the V_H_ homodimer. Considering the effects of the mutations on the structure and stability of MAK33 V_H_, this strong effect on association for the majority of the mutants might also be caused by the low stability and changes in secondary and tertiary structure of the MAK33 V_H_ variants as already described. As most of the conserved interface residues possess one or several interaction partners within the other variable domain (Table S1), a similar effect on association is expected as soon as one partner is mutated. This holds true for the interaction partners P44 (V_L_) and L45 (V_H_) as well as F98A (V_L_) and V37A (V_H_) where our analysis identified the most drastic effect on association for the point mutations P44A and L45A. Consequently, those V_H_ point mutations in MAK33 and 1HEZ with an apparent effect on association but lacking a corresponding effect for the mutant affecting the partner residue in V_L_ might be due to changes in secondary (Figs 2 b and 3b) and tertiary (Figs 2 d and 3d) structure of V_H_. For 1HEZ also V_H_ domain stability seems to impact the association since the two mutants compromised in association (V37A, L45A) show, besides 1HEZ W47A which exhibits a monomer-dimer equilibrium, a significantly decreased stability compared to 1HEZ wt (Table 2, Fig. 5b).

### Antigen binding of V_H_ and V_L_ is influenced by conserved interface residues

**Figure 7:**
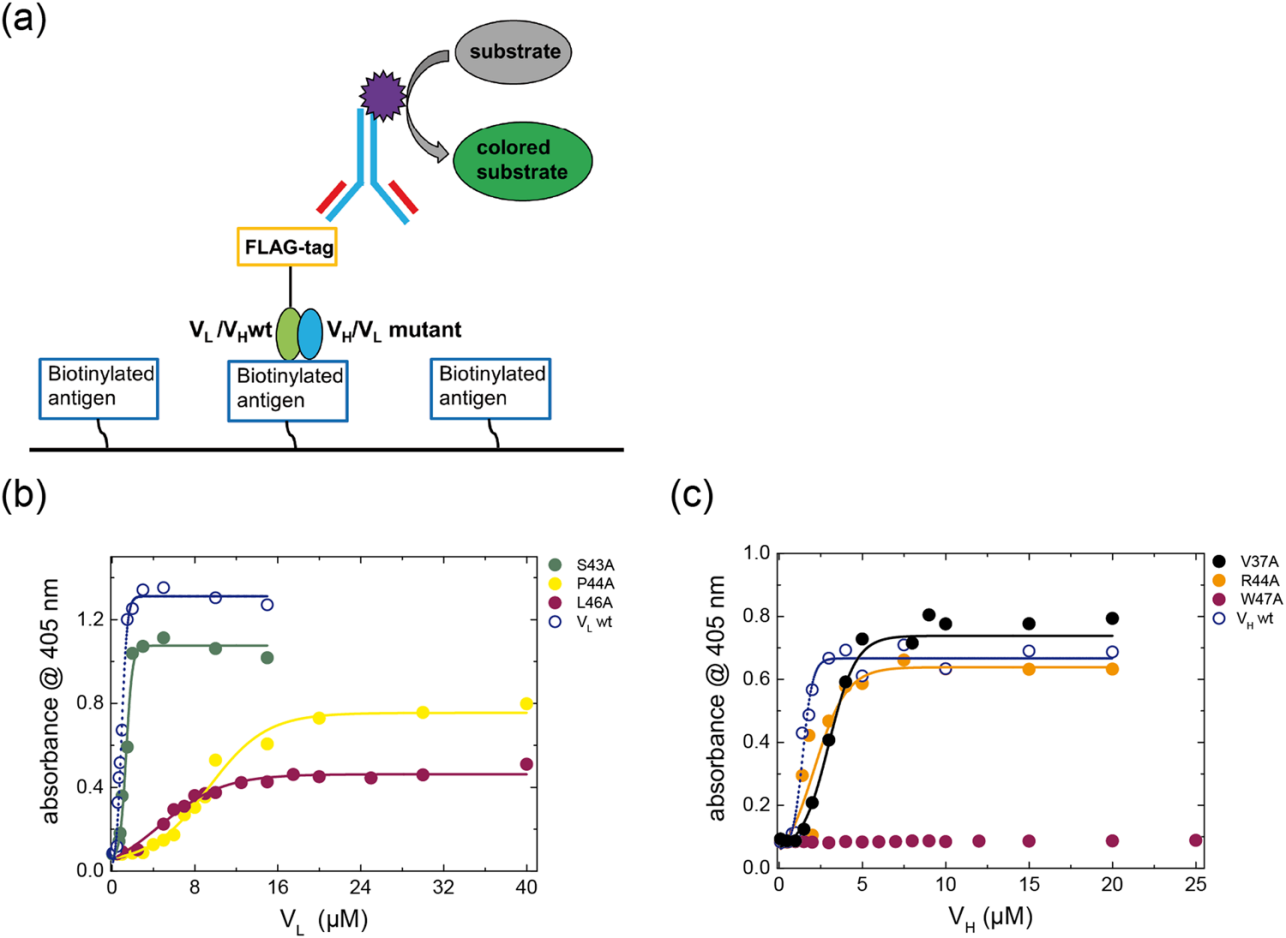
Influence of conserved residues on antigen binding. To determine the functionality of the mutants an ELISA with Fv fragments was performed. In (**a**) the ELISA setup is shown. The biotinylated antigen (creatine kinase) was immobilized on streptavidin coated 96 well plates. Subsequently, 1:1 mixtures of the FLAG-tagged wild type domain and a corresponding mutant were added. Binding was detected via a peroxidase-conjugated anti –FLAG antibody. The peroxidase mediated conversion of the substrate ABTS was measured colorimetrical at 405 nm. For V_L_ (**a**) and V_H_ (**b**), titrations of Fvs containing either the wild type, a representative of an inactive, a low active or a mutant with similar activity as the wild type is shown. The absorption at 405 nm at the signal maximum was corrected for the signal of the variable labelled domains alone. Experiments were performed at 20 °C.

To test how the mutation of conserved amino acids influences antigen recognition by the Fv fragment, we set up an ELISA (Fig. 7a) for the MAK33 Fv-fragment and human creatine kinase as its antigen. With the corresponding set up for the 1HEZ Fv fragment with an IgG Fc part as the antigen^29,30^, no binding was detectable. Hence, the influence of the conserved residues towards antigen binding could only be determined for the MAK33 Fv fragment. To this end, the respective wt domain was produced with a FLAG-tag at the C-terminus for detection with an anti-FLAG antibody. The tag does not negatively influence stability, folding and the interaction between the variable domains (data not shown). In this ELISA, a concentration-dependent increase in signal is only observed when V_L_ and V_H_ are combined (Fig. 7b and c). As the assay involves several protein interactions, only conclusions on an apparent K_D_ seem reasonable (Table 1). For the wt Fv with either V_L_ or V_H_ tagged with FLAG similar K_Dapp_s were determined (data not shown). Antigen binding was found to be abolished for V_H_ W47A. Apart from that, the V_H_ mutation L45A led to the strongest reduction in the affinity for the antigen with a 12-fold increased K_D_ compared to wt, while V37A and W103A exhibited a less pronounced decrease with a 2-fold higher K_D_ (Table 1). In general, for the V_H_ point mutations a low antigen binding activity correlates with a high K_D_ for association of the Fv-fragment (see Table 1). However, W47A, showed only a small decrease in the K_D_ but no binding to the antigen.

For the V_L_ domain, there is in most cases a correlation between the K_D_ for association and antigen binding, except for L46A which shows a wt-like association to V_H_ but an impaired antigen binding. The V_L_ mutations L46A, F98A, P44A, as well as the double mutant Y36A/P44A have a negative influence on antigen binding (Table 1), with a 5–12-fold increased K_D_. As already observed for V_H_/V_L_ association, P44A exhibited the weakest binding with a 12-fold higher K_D_ for the antigen. Interestingly, the double mutant Y36A/P44A showed only 50% of the impairment of P44A alone, so there must be a compensating effect of the Y36A mutation. This coincides with the data for the V_H_/V_L_ association (Table 1), where P44A exhibited the worst K_D_ while the double point mutation Y36A/P44A showed a 2-fold higher affinity than P44A alone. Surprisingly, V_L_ L46A and F98A, which exhibited an affinity for the V_H_ domain similar to the wt, were defective in antigen binding (Table 1). Consequently, the analysis of V_L_ mutants supports the assumption that the affinity between V_H_ and V_L_ is not necessarily correlated with the ability to bind the antigen.

### The CDR regions affect domain structure, stability and association

The CDRs of the antibody variable domains are elements of natural variations. How variations in these elements affect their association, structure and stability is therefore of special interest to obtain a comprehensive picture of the factors shaping the Fv-fragment. To address this question, we switched CDRs between MAK33 and unrelated variable domains. We chose human variable domain consensus sequences^31^ with CDRs of similar length. For V_L_, we selected the 1DH5 domain, and for V_H_ 1DHU. These human variable domains represent a class of variable domains with a highly stable structure^32^.

The K_D_s for the association of grafted variants with wt domains were determined by SPR. Due to the fact that the grafting constructs represent consensus sequences, no natural V_H_ or V_L_ partner domains exist. Hence, association was always determined with the corresponding MAK33 wt domain. For the association of the MAK33 V_H_ domain containing the 1DHU CDRs (1DHU_MAK V_H_) with MAK33 V_L,_ a K_D_ of 1.3 ± 0.1 µM was obtained. This corresponds to the value determined for the MAK33 wt domains (Table 1). Wt 1DHU V_H_ was a very good binding partner for MAK33 V_L_, with a K_D_ of 1.0 ± 0.5 µM. A similar value was measured when grafting the MAK33 CDRs into the 1DHU framework (MAK_1DHU V_H_), the chimera bound to MAK33 V_L_ with a K_D_ of 2.5 ± 0.3 µM. These observations lead to the conclusion that for MAK33 V_L_, the MAK33 and 1DHU V_H_ domains are both suitable binding partners. But when grafting the MAK33 CDRs on the 1DHU framework, the association is slightly impaired, demonstrating that the CDRs can exhibit a marked influence. Concerning the V_L_ grafting constructs, the observations are different: the 1DH5 V_L_ domain binds to MAK V_H_ with a roughly 30-fold lower affinity than the MAK33 V_L_ domain. However, the 1DH5 grafting construct containing the MAK33 CDRs (MAK33_1DH5) shows an affinity similar to the MAK33 wt V_L_ domain. In contrast, grafting of the 1DH5 CDRs onto the V_L_-MAK33 framework (1DH5_MAK33) resulted in strong binding impairment with a K_D_ of 42.3 ± 9.8 µM, which is only slightly lower than the 1DH5 wt K_D_ of 76.2 ± 12.8 µM. So for the V_L_ domain, the CDRs and not the framework are the determining factor for the affinity towards V_H_ wt.

**Figure 8:**
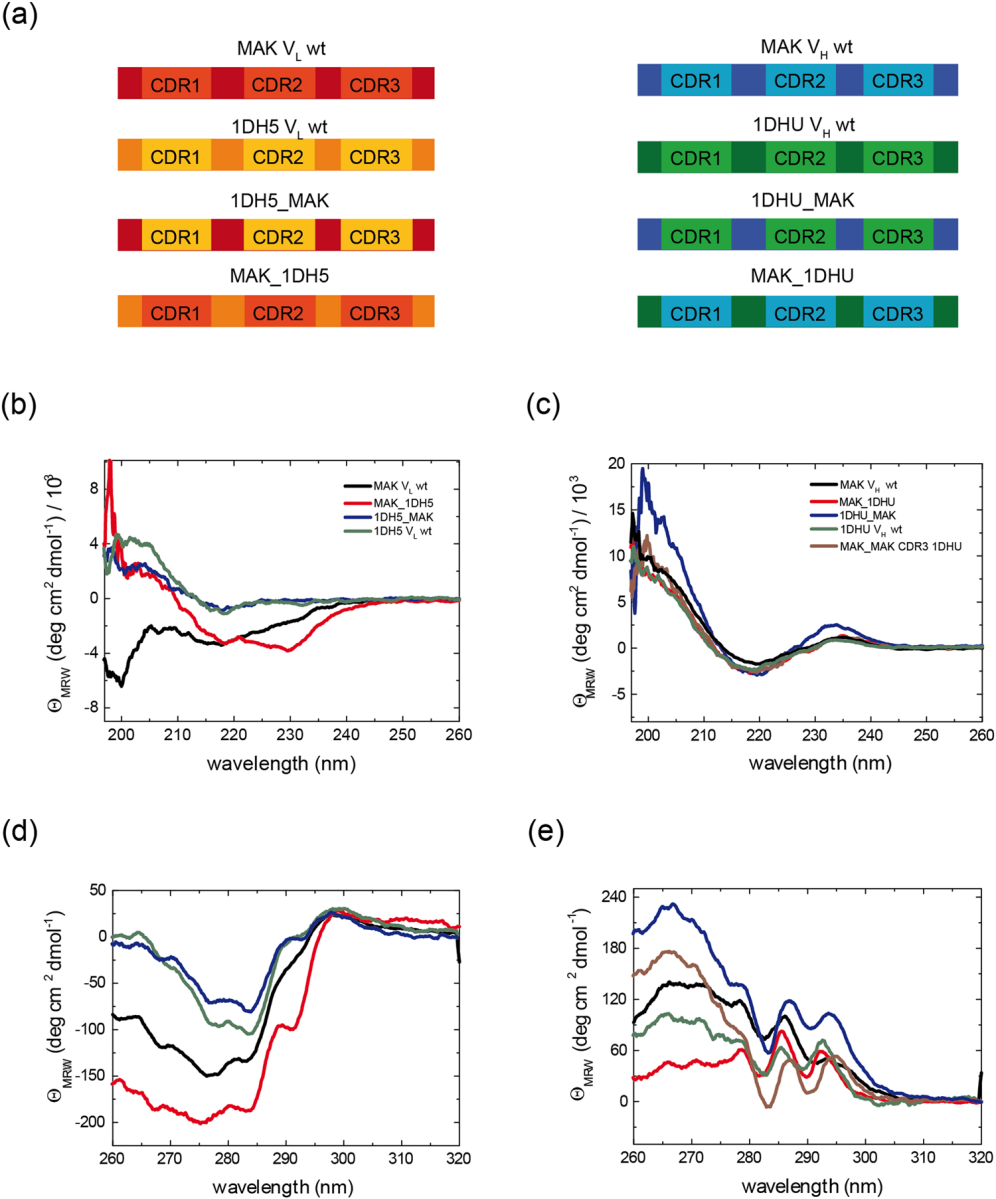
CDR-grafted mutants. (**a**) Schematic representation of CDR-grafted mutants. The framework for V_L_ grafting mutants (left) was 1DH5, which is the most stable human consensus sequence V_L_ domain (from HuCAL^31^). The framework for V_H_ grafting mutants (right) was 1DHU, which is the most stable human consensus sequence for V_H_ domains. In (**b** and **c**), FUV-CD spectra of V_L_ (left) and V_H_ (right) CDR-grafted mutants are shown. The color code for V_L_ is: MAK_1DH5 in red, 1DH5_MAK in blue; wild type 1DH5 is shown in green MAK33 in black. The color code for V_H_ is: MAK_1DHU in red, 1DHU_MAK in blue, wild type 1DHU in green, MAK33 in black and MAK33 CDR3 1DHU in brown. In (**d** and **e**) NUV-CD spectra of the V_L_ (left) and V_H_ (right) point mutants are shown.

The FUV and NUV spectra of the grafting mutants gave a similar picture. The CDR exchange (1DH5_MAK) showed a FUV-CD spectrum similar to 1DH5 V_L_ while the spectrum of MAK_1DH5 was different from both wts (Fig. 8b). So already on the secondary structure level the CDRs seem to be structurally important. The NUV-CD spectra exhibit a similar pattern (Fig. 8d) but here the difference in the number of aromatic amino acids, predominantly concerning the CDRs, could also play a role.

For the V_H_ domain, both grafting mutants showed similar FUV-CD spectra (Fig. 8c). The same was observed for the NUV-CD spectra of the grafting mutants compared to the wt domains; all spectra were similar in shape with variations in amplitude (Fig. 8e). As for V_L_ the observed NUV deviations might also be due to the different numbers of aromatic amino acids. For both domains the CDRs of MAK33 and 1DHU/1DH5 differ by one tryptophan and several tyrosines. Generally for MAK V_H_, the CDR exchange does not exhibit the same impact as for the V_L_ domain.

**Figure 9:**
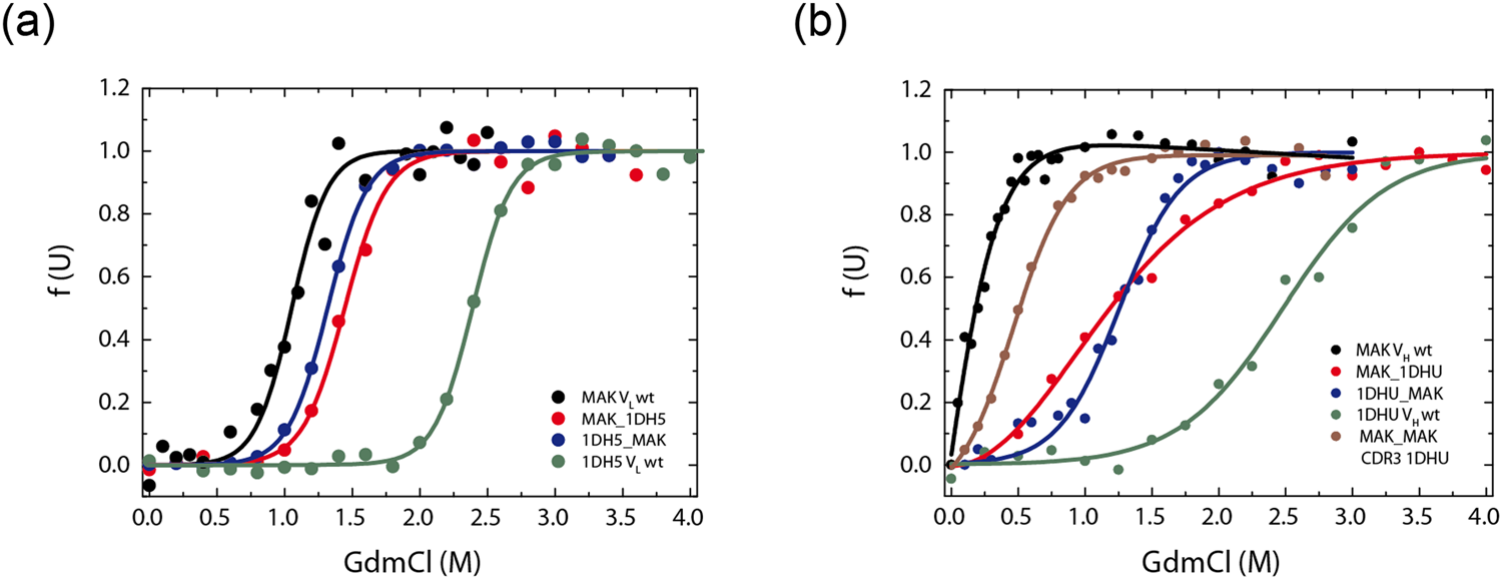
The influence of the CDRs on the stability of the variable domains. To assess the stability of CDR-grafted mutants, GdmCl-induced unfolding experiments were performed. Data for denaturant-induced transitions for V_L_ (**a**) und V_H_ (**b**) variants are shown. Data were evaluated according to a two-state unfolding model to obtain midpoints and cooperativity parameters of the transitions. Measurements were performed at 20 °C at a protein concentration of 1 μM.

GdmCl-induced transitions of the different V_L_ domains showed that, in comparison, the MAK33 V_L_ domain is least stable against chemical denaturation (Fig. 9a). As expected [19], the most stable domain was 1DH5 with a D_1/2_ of 2.4 ± 0.2 M. The grafted mutants showed stabilities in-between MAK33 V_L_ and 1DH5. Interestingly, the stability of the MAK33 V_L_ framework was increased when the CDRs were exchanged against the CDRs of 1DH5. On the V_H_ side, 1DHU showed the highest stability. The grafting mutants were again in-between (Fig. 9b). When the CDRs of 1DHU V_H_ were transplanted into MAK33, the chemical stability increased, however the transition was less cooperative compared to MAK33 V_H_ (Fig. 9b). Exchanging only CDR-H3 in MAK33 V_H_ led to a slight increase in stability compared to the wt (Fig. 9b). The exchange of the CDRs of 1DHU against MAK33 CDRs decreased its stability. In summary, the analysis of the chemical stabilities allowed the same conclusion for V_L_ and V_H_: the CDRs influence the stability of the variable domains decisively.

When antigen binding of the chimera was analyzed, the exchange of the MAK33 CDRs with that of the human consensus sequences led to a complete abolishment in both cases, as expected. For the grafting of MAK33 CDRs on the human variable domains, an interesting picture emerged. V_L_ (MAK_1DH5) has a slightly higher K_D_ of 0.3 µM for creatine kinase whereas there is almost no antigen binding detectable for the V_H_ grafting domain MAK _1DHU (>50 µM) (Table 1). So for binding of the V_H_ domain to the antigen not only the CDRs represent a determining factor.

As CDR-H3 (i.e. the third CDR of V_H_) is the most flexible of these six regions concerning length and amino acid sequence^2^, additionally a mutant carrying a swapped CDR-H3 loop was analyzed. Concerning the V_H_/V_L_ association the CDR-H3 mutation shows a wt-like K_D_ of 1.8 ± 0.2 µM. Interestingly, this construct, MAK CDR-H3 1DHU V_H_ showed a strongly impaired binding to the antigen with a 10-fold increased K_D_ (Table 1). This demonstrates the importance of CDR-H3 for the antigen binding of MAK33.

### Molecular Dynamics Simulations reveal mutation-induced structural alterations

For a subset of V_H_ and V_L_ mutations explicit solvent Molecular Dynamics (MD) simulations were performed for the heterodimer and for the individual domains. The set of simulations included point mutations that are associated with a significant reduction in V_H_/V_L_ association (V_L_ P44A, Y36A/P44A; V_H_ V37A, L45A and W103A) and, as a control, also substitutions that showed only modest effects on complex affinity (V_L_ Y36A, S43A; V_H_ R44A, W47A). Simulations were started from the geometry of the wt structure (pdb-entry: 1FH5, see Methods for details). On the time scale of the simulations, none of the V_H_/V_L_ complexes dissociated and the root-mean-square deviation (RMSD) of the complexes did not exceed 0.3 nm from the start structure (Fig. S1) and interface contacts as in the WT complex (Table S1). However, some of the mutations (e.g. V_L_ P44A, V_H_ R44A, V_H_ L45A and V_H_ W47A, see Fig. S1) resulted in overall larger final RMSDs compared to the wt indicating mutation-induced structural alterations and increased conformational freedom. Interestingly, simulations of the mutated proteins in the isolated state showed no significant differences in the RMSD (Fig. S2) except for V_H_ W103A (see below). The structural distortion of the V_L_/V_H_ complexes due to some of the mutations is also reflected in overall larger root mean square fluctuations of heavy atoms (RMSF) with respect to the mean structure. For the isolated mutated protein partners no significant difference to the wt was observed (Fig. S3), again with the exception of W103A.

**Figure 10:**
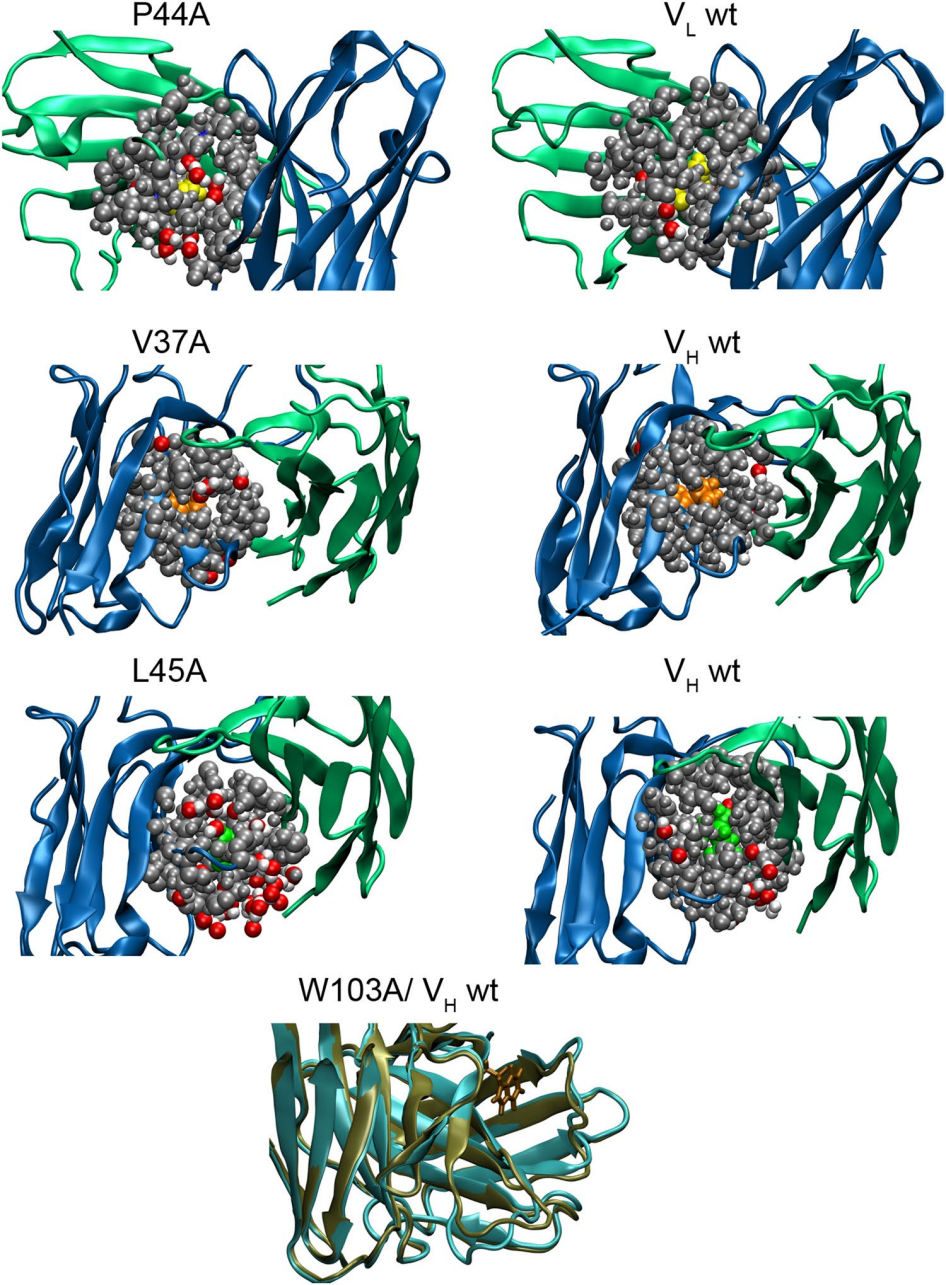
Interface hydration near mutation as determined by MD simulations. The snapshots illustrate the water distribution within 7 Å of the mutation site. Protein chains are shown as cartoon (V_L_: green, V_H_: blue). Atoms within 7 Å of the mutation site are indicated as van der Waals spheres using atom color code for water molecules (and grey for protein atoms). For comparison the same regions are also shown for the wild type case (left panels). In case of the V_H_ W103 mutation the average shift in backbone structure is illustrated (green cartoon) and compared to the wild type case (light blue).

However, in the complex the mutations with reduced binding affinity (e.g. V_L_ P44A, V_H_ R44A, V37A, L45A, W47A) showed increased fluctuations in regions at and near the binding interface but also in loops involved in antigen binding (Figs S4 and S5). Interestingly, especially for the substitutions that caused the largest drop in affinity between the V_L_ and the V_H_ domains an increased solvation at the interface (diffusion of water molecules into the space created by the introduction of a small Ala residue) was observed (illustrated in Fig. 10, Table 3). Especially for V_L_ P44A, V_H_ L45A and V_H_ W47A, the average number of water molecules increased near the mutation site (Table 3). The mutation V_H_ W103A resulted in significant changes of the backbone conformation around the mutation site, specifically the loop formed by residues 93–107 (Fig. 10), explaining the larger RMSD and RMSF observed for the isolated V_H_ domain in this case (Figs S2 and S3).

**Table 3:** Average number of water molecules around a point mutation (MAK33).

Mutation	<water> in mutation	<water> in wild type
V_L_ P44A	6.8 (2)	4.3 (1)
V_L_ S43A	14 (4)	15 (3)
V_L_ Y36A	1.1 (0.3)	0.8 (0.3)
V_H_ V37A	1.1 (0.4)	0.9 (0.3)
V_H_ R44A	16 (3)	15 (3)
V_H_ L45A	9 (3)	5 (2)
V_H_ W47A	8 (3)	5 (1)
V_H_W103	14 (3)	15 (3)

Water molecules within 7 Å of the C-beta atom of a mutated amino acid were determined during a 100 ns MD simulation. The standard deviation of the number of waters is given in parenthesis.

Besides of the effect of the mutations on the binding interface, it is interesting to investigate the changes in mobility of the CDR loops involved in antigen binding. We compared the fluctuation pattern observed in the complexes and in the individual (isolated) V_L_ and V_H_ partner domains. Even in case of the wt, the RMSF pattern changes significantly in several regions that include regions involved directly in binding the partner domain but also regions involved in antigen binding (Figs S3, S4 and S5). For example, the antigen binding loop V_H_: 93-107 shows large fluctuations in the absence of the V_L_ binding partner (Fig. S3) which drop significantly in the complex (Fig. S4). Hence, complex formation of the V_L_ and V_H_ domains appears to lock some of the antigen binding loops into distinct conformations. This effect is qualitatively also observed for several mutants, however, for some loop regions the reduction of CDR loop mobility upon binding is smaller compared to the wt. This is especially seen for the V_H_: 93–107 region.

In addition to simulations of point mutations, we also studied a subset of the loop exchange constructs (MAK_1DH5, 1DH5_MAK, MAK_1DHU and 1DHU_MAK). In experiments these variants affected the binding affinity between V_H_ and V_L_ domains much less than some of the interface point mutations (see above). During simulations on the time scale of 100 ns, these variants did not show significant differences of the calculated RMSF compared to the wt (Fig. S6).

## Discussion

The relationship between structure, stability and binding affinity of V_H_ and V_L_ is still unclear. This is an important aspect for understanding antibody architecture both as the basis of our immune system and also in the context of the engineering of antibodies for therapeutic purposes. In this context, it was found that in mutants an increase in affinity is often accompanied by a decrease in stability and *vice versa -* and these consequences are difficult to predict^33–39^. In our study, we analyzed the association of the variable domains for the first time directly. This allowed us to specifically define the contribution of framework and CDR mutations on the interaction of V_H_ and V_L_. To determine whether the observed effects for the conserved residues can be transferred between different variable domains, the analysis was performed with two distinct pairs of variable domains from a murine IgG1 (MAK33) and a human IgM (1HEZ) antibody. For the V_L_ wt domains, K_D_s of 2.0 ± 0.8 µM for the murine IgG1 and 8.2 ± 1.4 µM for the human IgM were determined. K_D_s for wild type V_H_ domains were found to be 2.9 ± 1.1 µM and 4.2 ± 1.1 µM for IgG1 and IgM, respectively. Consequently, the two domains interact with a similar efficiency as C_H_1 and C_L_, but not as efficient as the C_H_3 homodimer^19,21^. This relatively weak interaction (in the absence of the C_H_1 and C_L_ domains) necessitates the covalent linkage of Fv fragments via a peptide and thus creating a pseudo-monomeric fusion protein (scFv) used as a therapeutic agent^40,41^. Simulations of scFvs have shown that the stability of the interface between the two variable domains plays a critical role for the overall stability of an antibody (or fragment) as dissociation precedes unfolding^42^.

Of special interest is the nature of the domain interface. It has to support the association of the two domains but also allow accommodating different CDRs and their repositioning in the context of antigen binding. Thus, an individual interface residue may be involved in one or more of these processes: 1) formation of the immunoglobulin fold, 2) domain stability, 3) interaction between the variable domains, or 4) antigen binding. Our alanine screen of conserved residues allowed us to address each of these possibilities and differentiate between them. Consistent with the results of *in silico* analysis which showed that only very few residues (<10) are important for adopting an immunoglobulin fold^43^, the alanine mutants in the V_L_ domain had only a relatively low influence on its secondary or tertiary structure compared to the V_H_ domain. In contrast, V_H_ structure is highly sensitive to the exchange of conserved residues in the interface. Two V_H_ interface residues, E46 and R38, were identified to be essential for the folding of both, the MAK33 and 1HEZ V_H_ domain. In the covariation analysis there was a very high ɸ-value for these two residues. In the crystal structure, both residues form a salt bridge^9^, R38 is buried and E46 does not interact with other V_H_ interface residues, but might electrostatically affect V_L_ binding^9^. Camelids and cartilaginous fish possess naturally occurring heavy chain antibodies lacking the light chain^22,44,45^. Interestingly a sequence alignment of MAK33 V_H_ with the variable domain (V_HH_) of the camelid VHH (PDB entry 2XT1) and the variable domain of “monomeric” shark IgNAR (V_NAR_) (PDB entry 2I24) shows a match for the residues E46 and R38. Concerning the shark IgNAR these two residues are actually the only ones from the conserved network investigated in this study that are present at corresponding positions of this otherwise highly divergent sequence. For camelid V_HH_, a mutation of the hydrophobic V_H_/V_L_ interface residues (including the tetrad: V37, G44, L45, W47) in favor of hydrophilic ones was discovered^9,45^. The increase of hydrophilic residues in the framework also holds true for the V_NAR_ which probably evolved from a cell surface receptor^44,46^. Both antibodies show high biophysical stability and their distinct structural patterns have by now been successfully applied to generate monomeric human V_H_ domains^47–49^.

Mutations of conserved V_L_ residues had predominantly less impact than observed for V_H_ residues. As expected, MAK33 V_L_ S43A and MAK33 V_H_ R44A behaved similar to the wt. These are the only amino acid positions of the two antibodies studied which do not exhibit the conserved amino acid network identified by Wang and coworkers. In their covariation analysis an alanine is the conserved amino acid at V_L_ position 43 and glycine is conserved at position 44 in V_H_, as can be seen for the 1HEZ variable domains.

For both V_L_ domains, the proline residue at position 44 showed the most prominent effects with an impaired V_H_/V_L_ association as well as impaired antigen binding, followed by F98A. In contrast, P44 did not exhibit significant impact on V_L_ stability. Generally, a change in stability did not necessarily coincide with a change in functionality. Surprisingly, MAK33 V_L_ L46A exhibited a higher affinity for the V_H_ domain than the wt but it is clearly less efficient in binding the antigen (Table 1). The MAK33 double point mutations Y36A/P44A and Y36A/S43A indicate that the analyzed mutations can in principle compensate each others effects. In terms of stability, the stable mutation S43A is able to slightly improve the low stability of Y36A but, for the V_H_/V_L_ association, Y36A in combination with the worst binder P44A doubles the affinity for V_H_ compared to P44A alone. This coincides with the fact that these two residues are supposed to interact and are the most important V_L_ interface residues in the covariation analysis based on number and strength of covariations with other interface residues.

The biophysical properties of isolated V_H_ domains are in general more affected by mutations compared to V_L_^32^. MAK33 V_H_ domains show the lowest stabilities with a D_1/2_ value of 0.2 M GdmCl compared to 1.2 ± 0.1 M GdmCl for MAK 33 V_L_ and 0.9 ± 0.1 M for 1HEZ V_H_. But in the case of 1HEZ V_H_, it can be speculated that the higher stability might be caused by the homodimer formation, which is probably associated with a shielding effect of otherwise exposed hydrophobic V_H_/V_L_ interface residues. The homo-dimerization of 1HEZ VH variants (except W47A) adds another dimension to this study since it validates that the impact of highly conserved residues is preserved even in the presence of competing homo-dimerization. For the majority of the MAK33 V_H_ point mutations, especially L45A, an increased K_D_ for association with the V_L_ domain could be detected, which correlated in most cases with an impaired binding of the antigen. But V_H_ W47A, for example, showed no antigen binding while the V_H_/V_L_ association was only slightly decreased. 1HEZ V_H_ exhibited an impaired binding to V_L_ only for two mutants L45A and V37A. According to the simulation data for MAK33 (Table S1), the assigned V_L_ interaction partners of these two residues are P44 and F98. This fits perfectly to the identified impairments in V_H_/V_L_ association of the two V_L_ mutants in both V_L_ domains studied. Taken together, the MAK33 and 1HEZ data for the V_H_/V_L_ association point toward a striking importance of the interaction partners L45(V_H_)/P44(V_L_) and a contribution of V37(V_H_)/F98(V_L_). In terms of antigen binding, it has to be kept in mind that additionally to the stabilizing effect of the V_L_ domain, antigen binding represents for the heterodimer an additional layer of stabilization. For the MAK33 V_H_ domain, in isolation some of the conserved V_H_/V_L_ interface residues are critical for structure formation and stability. This might cause the impaired association with the V_L_ domain. In general, association and antigen binding do not necessarily correlate.

Surprisingly, we could not find a general consistent relationship between secondary and/or tertiary structural changes, stability, association or antigen binding. Since it is known that antibody domains share the common ‘Ig fold’, the mutations most likely do not alter the highly conserved Ig fold, but rather lead to local structural changes in agreement with the MD simulations. Due to V(D)J joining and somatic hypermutations, immunoglobulin variable domains naturally experience an extreme sequence variation, except for some rare highly conserved residues. It thus makes sense that the Ig fold has an intrinsically high tolerance to structural changes. Interestingly, our results show that this is also the case for functionally important residues. However, in some cases natural mutations can disrupt the stable Ig topology, leading to the rare but severe disease AL amyloidosis^27^.

It seems that during antibody biogenesis the effect of CDRs on the stability of V_H_ domains is a decisive, so far underappreciated factor. Especially concerning the observed MAK33 V_H_ instability, the outcome of grafting experiments with stable human consensus sequences was interesting. The grafting constructs revealed that CDRs, in addition to antigen binding, affect variable domain structure strongly. This is especially true for CDR-H3 (Table 1). Comparing the MAK33 and 1DHU/1DH5 CDRs, CDR- H3 differed most, in terms of length (14 amino acids for MAK33 versus 11 for 1DHU) as well as charge. According to Morea and coworkers, CDR-H3 conformation does not only depend on the environment^2^ but can be assigned to different conformation types based on some key amino acid positions in the CDRs. Since MAK33 possesses a lysine at position 94 and an aspartate at position 101, which can form a salt bridge, it is assumed to have a bulged conformation in contrast to 1DHU. As the CDR-H3 also contributes to the interface and interacts with the V_L_ domain, this conformational difference might not only affect antigen binding. In our case, the exchange of the MAK33 CDR-H3 impairs antigen binding (10-fold increased K_D_) and slightly increases domain stability. The data for the grafting constructs show a very different picture for the V_H_ and V_L_ domain. For V_L_, the CDRs seem to be important for structure and V_H_/V_L_ association but concerning antigen binding, also within the 1DH5 framework, the affinity is wt-like. The V_H_ domain, though, did not exhibit such a strong CDR dependence for V_H_/V_L_ association, only the framework had a slight effect. However, antigen binding was almost not detectable when grafting the CDRs to the 1DHU framework. Since exchanging only CDR-H3 of the V_H_ MAK33 domain leads to a severe impairment of the antigen binding, this indicates a crucial role of the V_H_ domain and especially the CDR-H3 in this process. An explanation for these observations could be the mentioned differences between MAK33 and 1DHU V_H_ CDR-H3. Interestingly, grafting either the CDRs or framework from the human consensus sequences on MAK33 V_L_ or V_H_, always led to an increase in stability. This might be an important aspect for CDR selection and the interplay with domain stability. Previous studies applying CDR graftings for antibody humanization approaches showed the importance and complexity of the influence of specific framework residues in the context of antigen binding and stability improvement^11,36,50,51^. Here we could show that vice versa, CDRs themselves can be considered as a crucial determinant of stability.

Our MD simulations for the MAK33 variable domains indicate that the mutations altered conformational fluctuations of the isolated domains which cause structural and mobility changes at the binding interface. The non-optimal packing at the protein-protein interface leads to increased fluctuations at the interface which is also manifested in a reduction of interactions (reduced binding affinity) and also to fluctuations in the antigen binding loop regions which can reduce the binding affinity for antigens, in agreement with previous findings where it was shown that subtle changes in the interface can affect the affinity for the antigen^50^. Interestingly, we found both for the wild type but also for the mutants that regions involved in antigen binding change the flexibility pattern upon complex formation. Hence, the V_L_/V_H_ association appears to lock some of the antigen binding loops into distinct conformations and associated flexibilities. This is in line with the observed subtle influence of some interface residues on antigen binding affinity despite the location distant from the antigen binding region. In some cases also increased solvation at the interface was observed. This was especially the case for the mutations with the most impaired V_H_/V_L_ association (V_L_ P44A, V_H_ L45A). The presence of water molecules at the interface reduces intermolecular contacts between protein partners by giving the interface an increasing non-specific character. Thus, the experimentally observed changes in binding affinity and stability of the mutations are due to a combination of effects. A quantitative correlation with the experimentally observed change in antigen binding affinity due to the mutation is, however, not observed. It should be emphasized that this is also not expected since binding to the antigen is affected by each CDR loop differently and changes in loop mobility can in principle affect antigen binding affinity in an unpredictable way.

Taken together our data indicate that multiple determinants regulate the V_H_/V_L_ association and the affinity for the antigen. The interplay between interface interactions and CDRs turned out to be complex with mutual influences on V_H_/V_L_ association and antigen binding.

## Material and Methods

Unless otherwise stated, all experiments were carried out at 25 °C. Measurements were performed in 50 mM sodium phosphate buffer at pH 7.5

### Cloning and protein expression

V_H_ and V_L_ were cloned into pET28 A (Novagen, Darmstadt,Germany) with NcoI and HindIII (NEB, Hitchin, UK) and expressed in E. coli BL21 star (Invitrogen, Carlsbad, USA). The transformed cells were grown in LB medium containing kanamycin at 37 °C until an OD_600_ of 0.6-0.8 was reached. The expression was induced by the addition of 1 mM isopropyl β-D-thiogalactopyranoside (IPTG). After 12 h, cells were harvested, and preparation of inclusion bodies was carried out as described previously^23^. The purification was performed according to the procedure described for the V_L_ domain^24^. Protein purity was checked by SDS-PAGE (Fig. S7).

Single point mutations were introduced by a quick change PCR approach using the QuikChange® Site-Directed Mutagenesis Kit (Agilent Technologies Inc., Santa Clara, USA) according to the manufacturer’s recommendations. Primers were ordered from MWG Operon (Ebersberg, Gemany). Intact protein was verified by matrix-assisted laser desorption/ionization time-of-flight mass spectrometry.

### CD and Fluorescence spectroscopy

CD measurements were carried out using a Jasco J-720 spectropolarimeter (Jasco, Grossumstadt, Germany) equipped with a Peltier element. Far-UV CD spectra were measured using 10 µM protein in 1.0-mm quartz cuvettes between 260 nm and 198 nm and near-UV CD spectra between 320 nm and 260 nm using 50 µM protein in 5-mm quartz cuvettes. The spectra were accumulated 16 times and buffer corrected.

For denaturant-induced unfolding transitions, structural changes were monitored by fluorescence spectroscopy at 355 nm. Excitation wavelength was 280 nm and slit widths were 1 nm (excitation) and 3 nm (emission) for V_H_ and 2 nm and 5 nm for V_L_, respectively. All measurements were performed with 1 µM protein in a 1-cm quartz cuvette. The samples were incubated overnight at 20 °C at the different GdmCl concentrations prior to measurements.

Data evaluation was performed with Origin 8 G (OriginLab, Northampton, USA); for GdmCl-transitions a two-state model was applied^52^.

### SPR

V_H_/V_L_ association was measured by SPR experiments, performed with a Biacore ×100 (GE Healthcare, Freiburg, Germany). The wild type domain was immobilized on a CM5 chip by amine coupling chemistry. Subsequently, multi cycle runs with titrations of the mutant domains, ranging from 0–200 µM, were measured at 20 °C with an injection time of 60 seconds and a constant flow rate of 10 µl/min. After each cycle, bound protein was removed with 2 M NaCl regeneration solution.

### ELISA

Binding of the variable domains of MAK33 to the antigen creatine kinase was analyzed by ELISA. Assay components and microwell plates were from Roche (Mannheim, Germany). Samples were prepared in 10 µl volume. Different mutants were tested within a concentration range of 100 nM up to 50 µM against wild type V_L_ or V_H_ with a C-terminal Flag-tag for detection. After the addition of 90 µl reaction mix I, the sample was incubated in a streptavidine-coated microwell plate to immobilize human biotinylated creatine kinase. Incubation was performed for 45 minutes with constant agitation at 20 °C or 10 °C for V_L_ and V_H_ mutants, respectively. Afterwards, the samples were washed with sterile pure water for three times. Then 100 µl/well of reaction mix II were added together with the detection antibody for the Flag-tag coupled to horseradish peroxidase in a 1:15.000 dilution. Afterwards, the samples were washed three times with water again and then 100 µl of reaction mix III was added. The product of the enzymatic reaction was monitored at 405 nm in a GENios plate reader (Tecan, Männedorf, Switzerland) for 0.5–3 hours until a plateau was reached.

### Molecular dynamics simulations

Start structures of MAK33 V_L_/V_H_ complexes as well as individual V_L_ and V_H_ domains were obtained by extracting the corresponding coordinates from the crystal structures 1FH5^50^. Missing residues were added/corrected using the program PyMol^50^ with a final sequence corresponding exactly to the wild type sequence used in the experiments. Model start structures of all mutants were generated based on the wild type structure with residue substitutions generated *in silico*. All Molecular Dynamics (MD) simulations as well as the analysis of root-mean square deviation (RMSD) and fluctuations (RMSF) were performed using the Gromacs4.6 package^53,54^ in combination with the AmberSB99_ILDN force field^55^. Proteins were solvated in dodecahedral boxes including explicit ions (Na^+^ and Cl^−^) and explicit (TIP3P) water molecules^56^. The simulation systems were first energy-minimized (until the maximum force was smaller than 500 kJ/mol) followed by heating up to 300 K at a constant volume with position restraints on the protein. Subsequently, a pressure equilibration at 1 bar with position restraints on the protein was carried out. All production simulations were performed at a temperature of 300 K and a pressure of 1 bar and extended to 100 ns. Root mean square deviation (RMSD) and root mean square fluctuations (RMSF) with respect to the mean structure were calculated with g_rms and g_rmsf modules of Gromacs. Snapshots were created using VMD^57^.

The datasets generated during and/or analyzed during the current study are available from the corresponding author on reasonable request.

## Electronic supplementary material

Supplementary Information
