# Supplementary material for: Determinants of the assembly and function of antibody variable domains
Source: Sci Rep. 2017 Sep 25;7:12276. doi: 10.1038/s41598-017-12519-9 (PMC5613017; doi:10.1038/s41598-017-12519-9)
Supplement: Supplementary file 1 — Supplementary Information [file 41598_2017_12519_MOESM1_ESM.pdf]

Supplementary Information

Determinants of the assembly and function of antibody variable domains

Eva Maria Herold[1,2+], Christine John[1+], Benedikt Weber[1], Stephan Kremser[3], Jonathan Eras[4], Carolin Berner[1], Sabrina Deubler[1], Martin Zacharias[3] and Johannes Buchner[1,5]

[1] Center for Integrated Protein Science Munich (CIPSM) at the Department Chemie, Technische Universität München, 85747 Garching, Germany

[2] present address: Sanofi-Aventis GmbH, Industriepark Höchst, 65926 Frankfurt am Main, Germany

[3] Center for Integrated Protein Science Munich (CIPSM) at the Physics Department, Technische Universität München, 85747 Garching, Germany

[4] present address: ETH Zürich, Otto-Stern-Weg 5, 8093 Zuerich Switzerland

[+]these authors contributed equally

Supplementary Figures

Figure S1

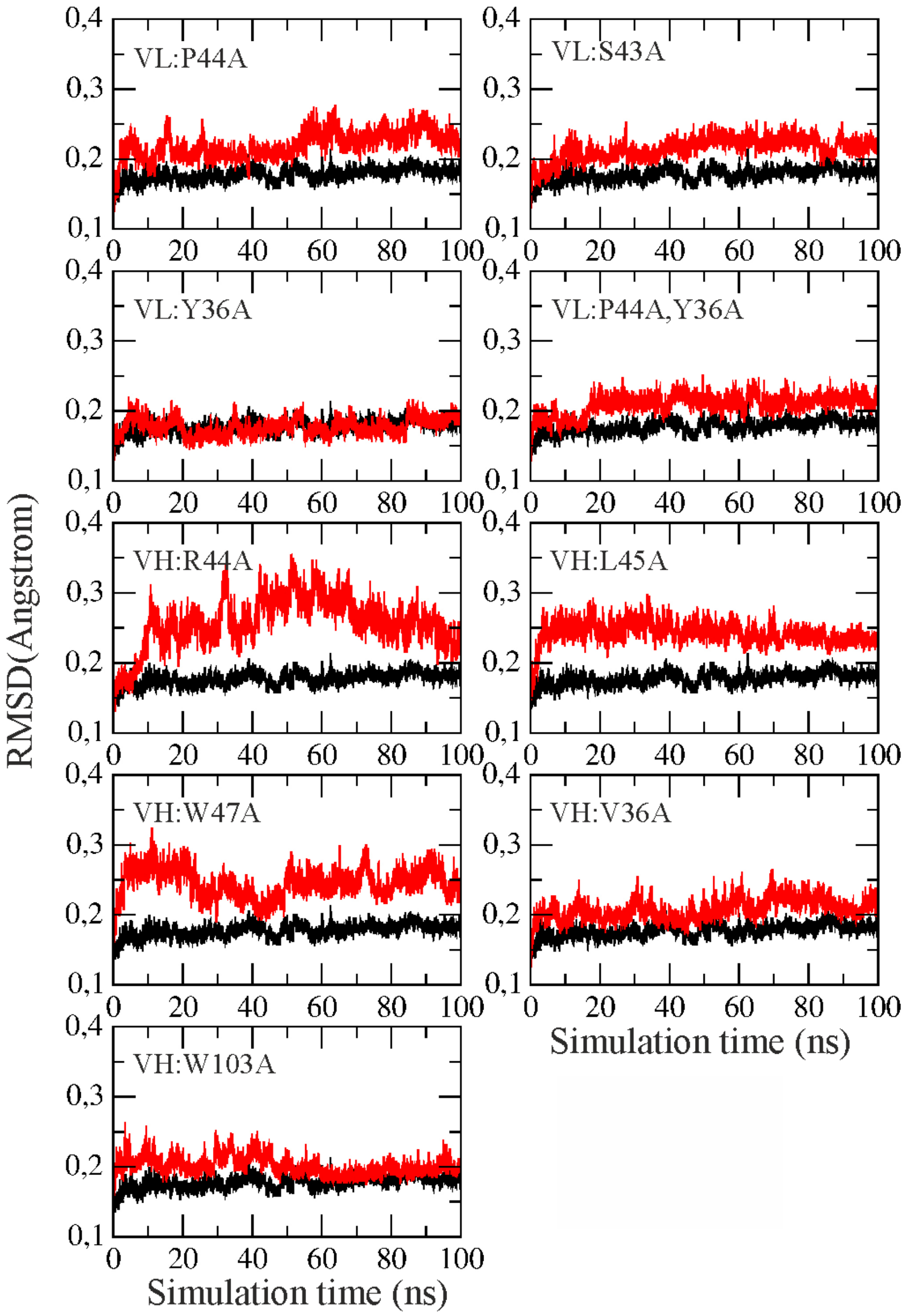

Figure S1. Root-mean-square deviation (RMSD) of the protein backbone (V_L/V_H complex) from the experimental start structure vs. simulation time. The RMSD recorded for the wild type complex is indicated in black (the RMSD of each mutation is shown in red).

Figure S2

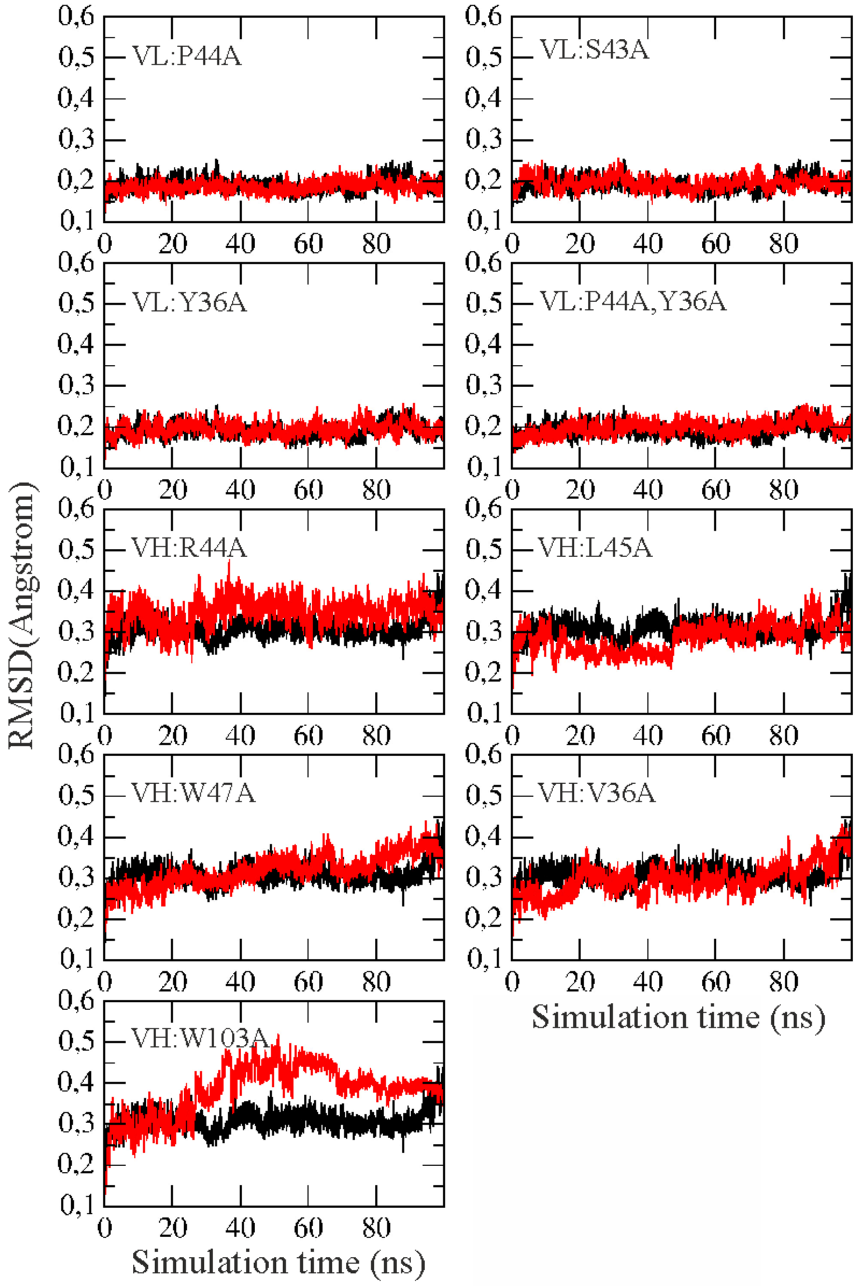

Figure S2. Root-mean-square deviation (RMSD) of the protein backbone of isolated V_L or V_H protein partners from the experimental start structure vs. simulation time. The RMSD recorded for the wild type protein domain is indicated in black (the RMSD of each mutation is shown in red).

Figure S3

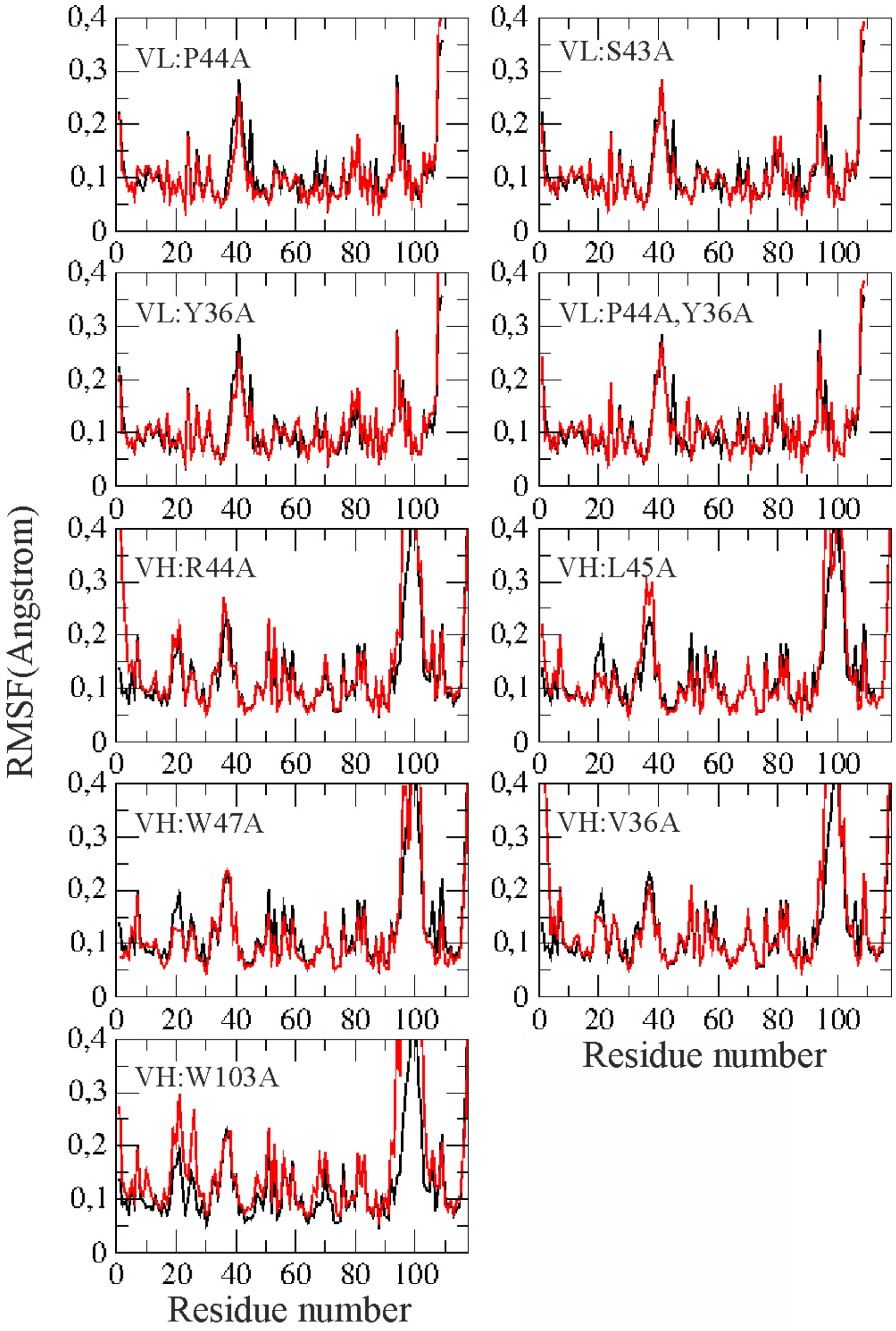

Figure S3. Root-mean-square fluctuation (RMSF) with respect to the mean protein structure during each 100 ns simulation. The RMSF was calculated for all atoms of a residue and plotted vs. residue number. The RMSF obtained for the wild type V_L or V_H domain is indicated in black (the RMSF of each isolated domain mutation is shown in red).

Figure S4

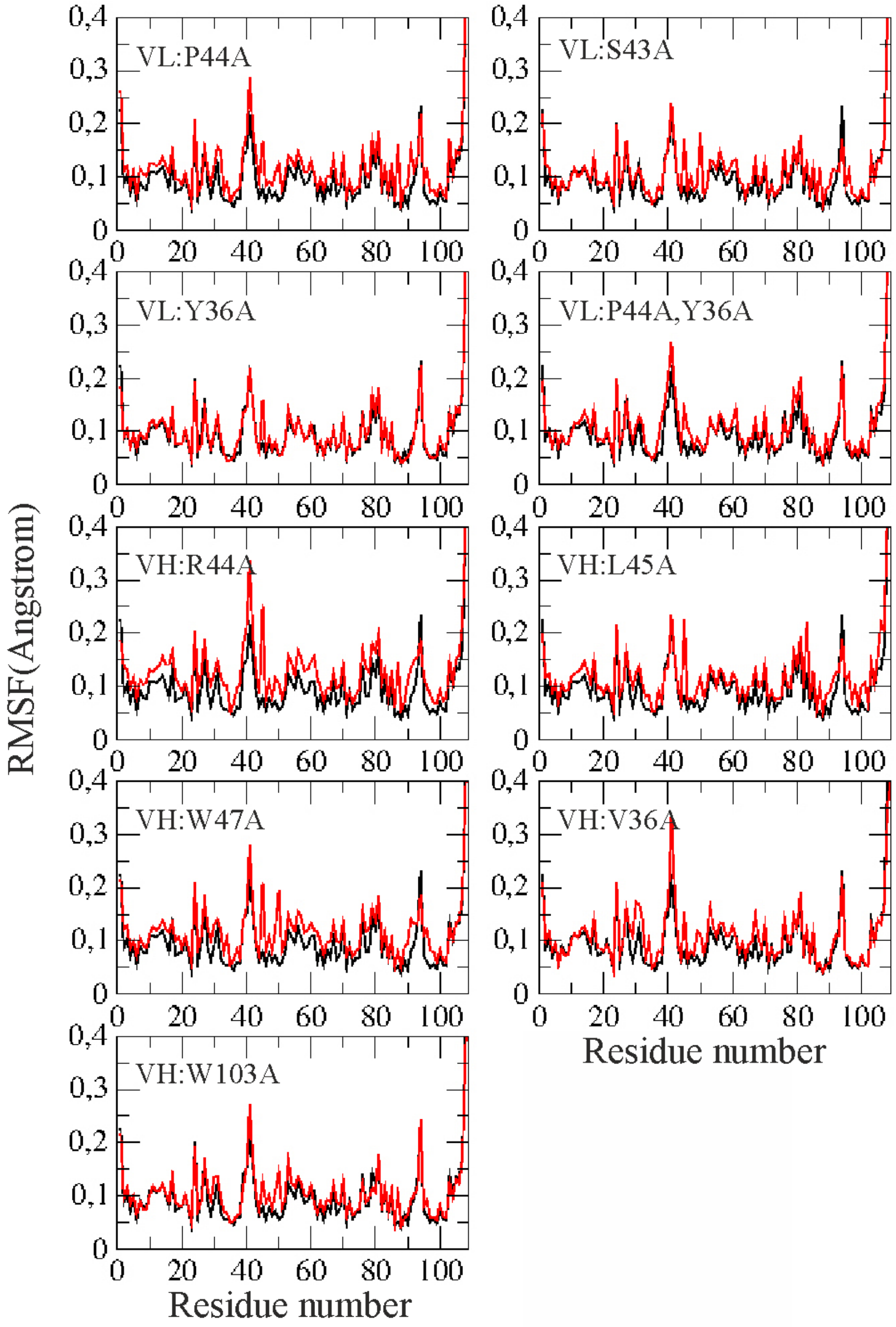

Figure S4. Root-mean-square fluctuation (RMSF) with respect to the mean V_L/V_H complex structure during each 100 ns simulation. The RMSF was calculated for all atoms of a residue and plotted vs. residue number of the V_L domain. The RMSF obtained for the wild type V_L domain (in complex with the V_H) is indicated in black (the RMSF of each isolated domain mutation is shown in red).

Figure S5

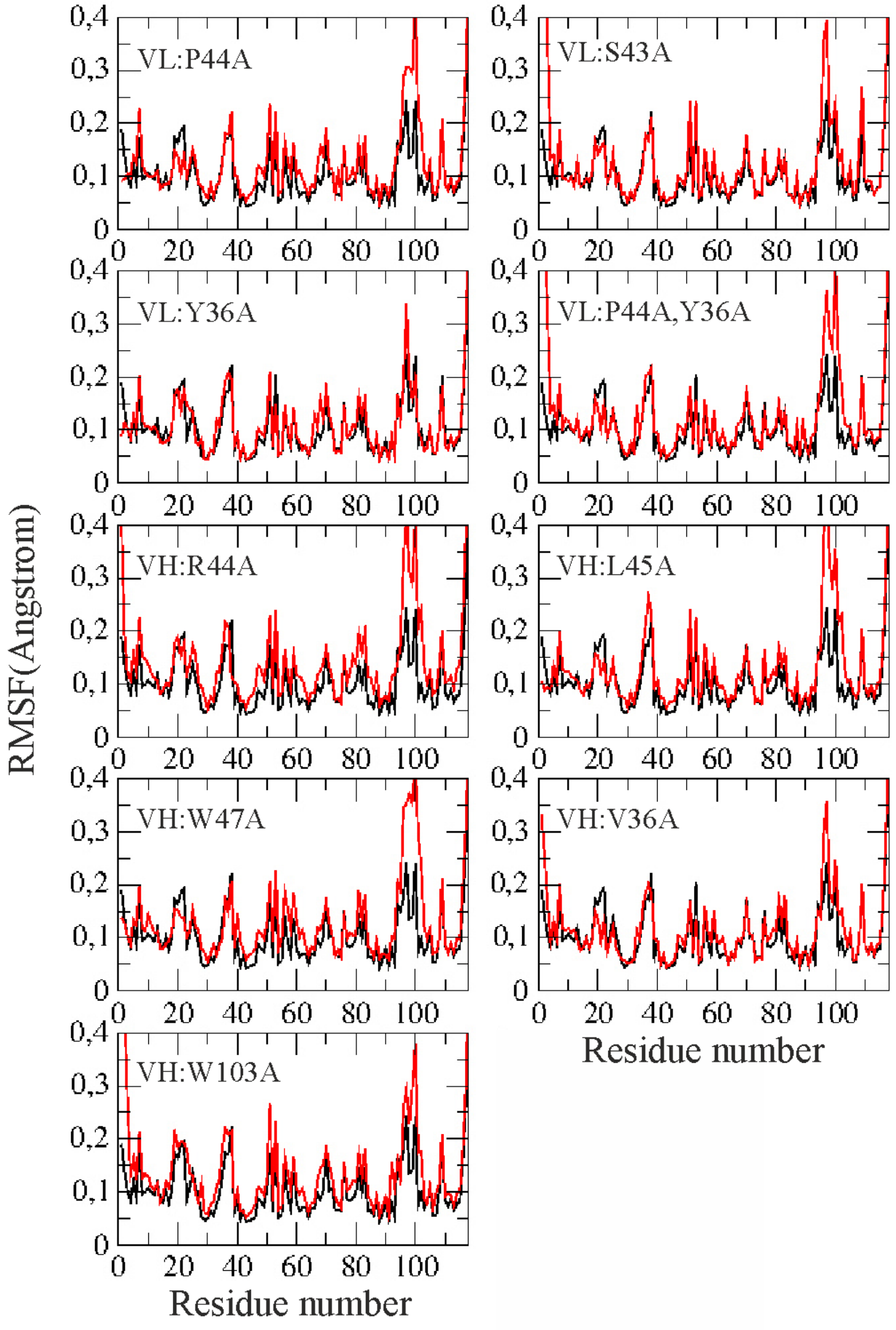

Figure S5. Root-mean-square fluctuation (RMSF) with respect to the mean V_L/V_H complex structure during each 100 ns simulation. The RMSF was calculated for all atoms of a residue and plotted vs. residue number of the V_H domain. The RMSF obtained for the wild type V_H domain (in complex with the V_H) is indicated in black (the RMSF of each isolated domain mutation is shown in red).

Figure S6

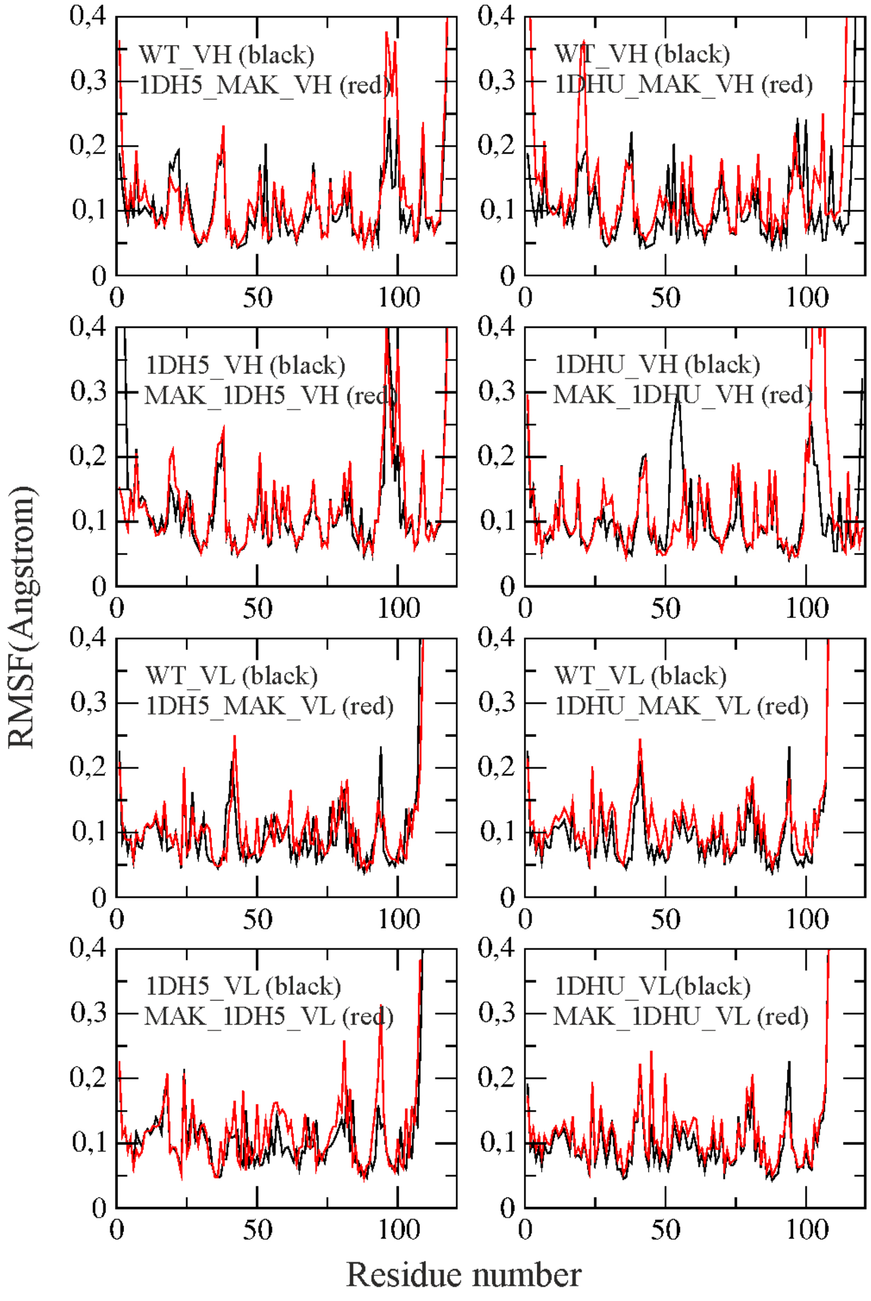

Figure S6. Root-mean-square fluctuation (RMSF) with respect to the mean V_L/V_H complex structure during each 100 ns simulation. The RMSF was calculated for all atoms of a residue and plotted vs. residue number of the V_H domain (upper four panels) and for the V_L domain (lower four panels). The RMSF obtained for the wild type case is indicated in black and for the loop exchange mutations in red.

Figure S7

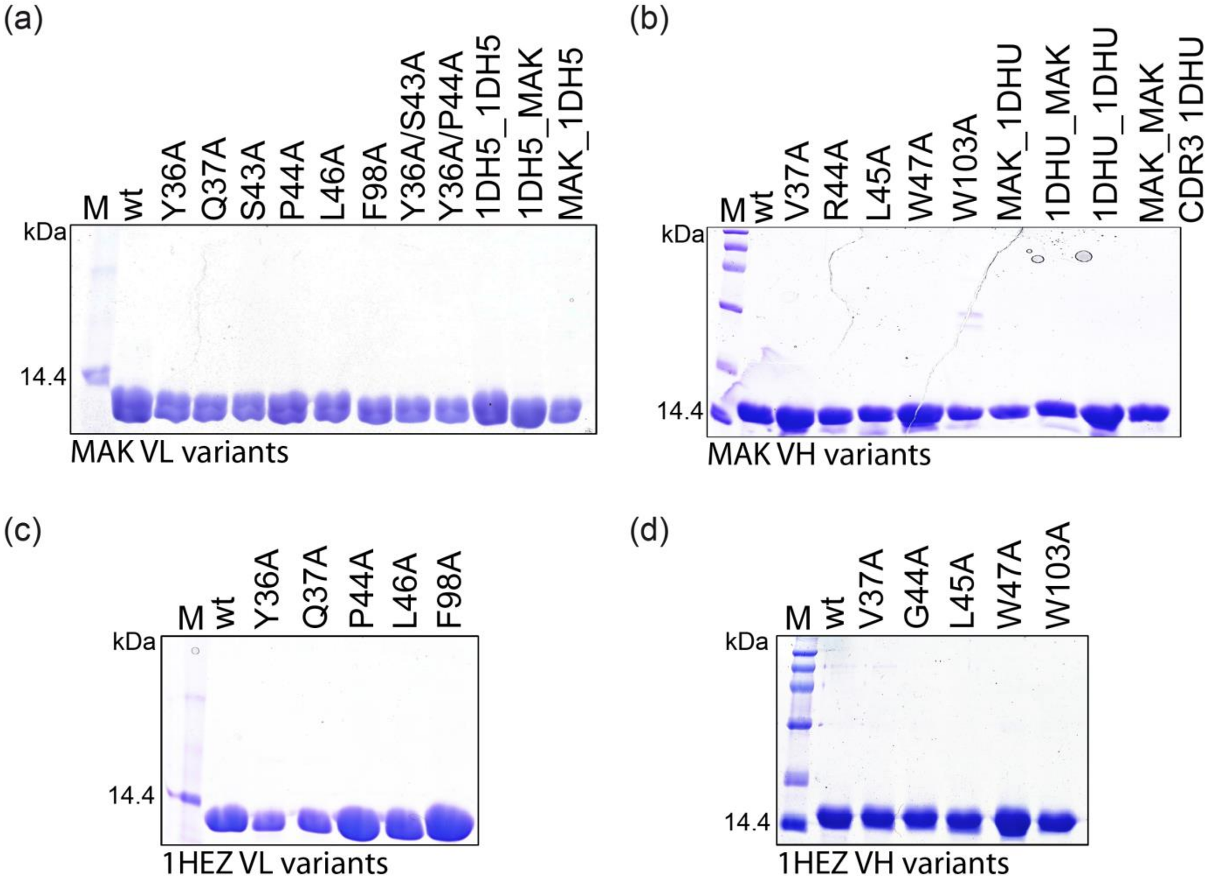

Figure S7. SDS-PAGE analysis of purified proteins. All proteins employed in this study were recombinantly expressed, refolded and purified as described in the methods section.

Figure S8

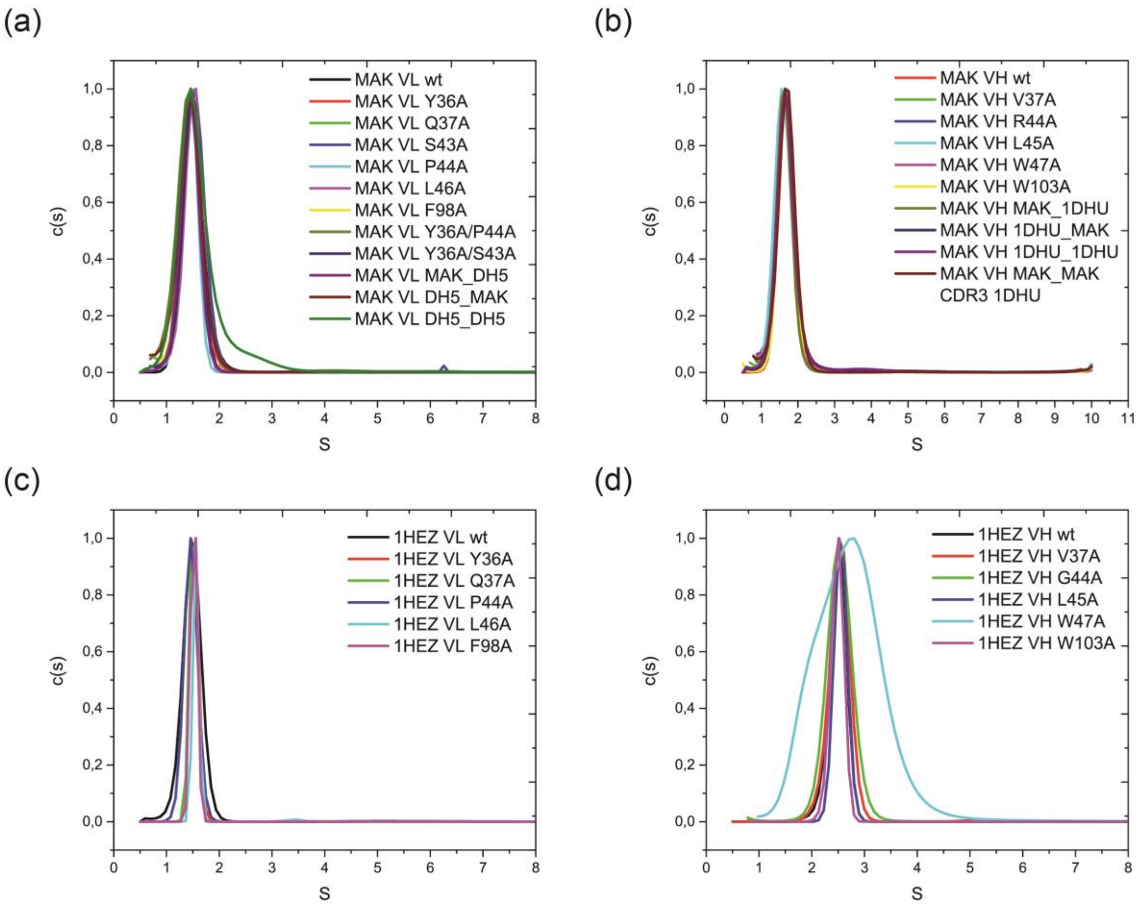

Figure S8. Analytical ultracentrifugation (AUC) plots of MAK VL (a), MAK VH (b), 1HEZ VL (c) and 1HEZ VH (d) variants. 1HEZ VH variants exhibit a dimeric quaternary structure except 1HEZ VH W47A which shows an equilibrium between monomers and dimers. All other proteins are monomers. Sedimentation velocity (SV) AUC experiments were carried out with a ProteomLab XL-I (Beckman, Krefeld, Germany) supplied with absorbance optics. All experiments were performed using PBS at 20°C as a reference. 350 µl sample were loaded into assembled cells with sapphire windows and 12 mm path length charcoal-filled epon double sector centerpieces and centrifuged at 42000 – 48000 rpm in an eight-hole Beckman-Coulter AN50-Ti rotor. Sedimentation was monitored with an UV/VIS spectrophotometer, equipped with a monochromator, at 260 nm. Data analysis was carried out with Sedfit (Peter Schuck, National Institute of Health, Bethseda, Maryland, USA), using a non-model based continuous Svedberg distribution method (c(S)), with time (TI) and radial (RI) invariant noise on.

Table S1. Interface contacts of residues analyzed in MD simulations

residue in V_L domain	contact in V_H domain
P44	Q39, L45, Y91, Y102
S43	Y91, Y102, (W103, G104, Q105)
Y36	LEU45, Y102, D101, M100F, A100E
**residue in V_H domain**	**contact in V_L domain**
R44	F87, (F98), A100
L45	P44, (Q37), F87, F98
W47	(W94), P95, L96, (T97), F98
V37	F98
W103	(S43)

Atom-atom distances < 5 Å between any side chain atoms in V_H and V_L domains during >90% of MD simulation time of the WT complex were counted as contact (residues in parenthesis showed contacts for 50-90% of the simulation time).
